# Transcriptome profiling in response to Kanamycin B reveals its wider non-antibiotic cellular function in *Escherichia coli*

**DOI:** 10.3389/fmicb.2022.937827

**Published:** 2022-11-29

**Authors:** Yaowen Chang, Xuhui Zhang, Alastair I. H. Murchie, Dongrong Chen

**Affiliations:** Fudan University Pudong Medical Center, and Institute of Biomedical Sciences, Shanghai Medical College, Key Laboratory of Medical Epigenetics and Metabolism, Fudan University, Shanghai, China

**Keywords:** Kanamycin B, transcriptome, aminoglycosides, differentially expressed genes, binding

## Abstract

Aminoglycosides are not only antibiotics but also have wider and diverse non-antibiotic cellular functions. To elucidate the understanding of non-antibiotic cellular functions, here we report transcriptome-profiling analysis of *Escherichia coli* in the absence or presence of 0.5 and 1 μM of Kanamycin B, concentrations that are neither lethal nor inhibit growth, and identified the differentially expressed genes (DEGs) at two given concentrations of Kanamycin B. Functional classification of the DEGs revealed that they were mainly related to microbial metabolism including two-component systems, biofilm formation, oxidative phosphorylation and nitrogen metabolism in diverse environments. We further showed that Kanamycin B and other aminoglycosides can induce reporter gene expression through the 5′ UTR of *napF* gene or *narK* gene (both identified as DEG) and Kanamycin B can directly bind to the RNA. The results provide new insights into a better understanding of the wider aminoglycosides cellular function in *E. coli* rather than its known antibiotics function.

## Introduction

Aminoglycosides are antibiotics that are known to inhibit translation by binding to the A site of the ribosome during the translocation process (Davies and Davis, 1968; Fourmy et al., 1996; Carter et al., 2000), including Kanamycin B, Gentamicin, Amikacin, Tobramycin, Neomycin, and Streptomycin etc. Aminoglycosides are broad-spectrum antibiotics that are potent against various Gram-positive and Gram-negative organisms including members of the *Enterobacteriaceae* family such as *Escherichia coli* (*E. coli*) (Krause et al., 2016).

There have been reports on the response and effect of aminoglycosides in *E. coli* and in other bacteria. Biofilms are bacterial aggregates that resist antibiotic treatment. Subinhibitory concentrations of aminoglycosides were shown to regulate biofilm formation in *E. coli* and also in *Pseudomonas aeruginosa* (Hoffman et al., 2005; Khan et al., 2020). Gentamycin at sub-inhibitory concentrations can inhibit the swarming motility of *E. coli* (Zhuang et al., 2016). In *E. coli*, it is known that the uptake of aminoglycosides requires proton motive force generated by electron flow through the respiratory chain (Complexes I and II containing Fe-S clusters). Fe-S proteins play an important role in aminoglycoside killing through their effect on aminoglycoside uptake (Ezraty et al., 2013). In some organisms the aminoglycosides are linked to the bacterial SOS response. For example, aminoglycosides were found to induce SOS responses in *Vibrio cholerae*. However, in *E. coli*, aminoglycosides was shown not to induce SOS (Baharoglu and Mazel, 2011).

Genome-wide analysis of transcriptome profiling of *E. coli* and other bacteria upon treatment with specific aminoglycosides have been reported. Upon treatment with subinhibitory dose of gentamicin (1/2 MIC) that aims to find genes that may be involved in the development of adaptive resistance, transcriptome data of *E. coli* has revealed that genes associated with membrane protein and transporter were highly regulated. The transcriptome data identified yhjX (a putative transporter) as most upregulated gene and it regulates the cell growth of *E. coli* at subinhibitory dose of gentamicin and associate with the adaptive resistance to gentamicin (Zhou et al., 2019). Transcriptome profiling of *Pseudomonas aeruginosa* upon treatment with Tobramycin revealed expression changes in genes involved in a wider cellular function such as amino acid catabolism, tricarboxylic acid cycle (TCA) or bacterial motility and attachment etc. (Cianciulli Sesso et al., 2021). RNA-Seq analysis of *Pseudomonas aeruginosa* swarming cells at subinhibitory concentrations of tobramycin identified expression changes in gene encoding multidrug efflux pump and virulence factors (Coleman et al., 2020). In addition, transcriptional profiling changes in *Mycobacterium tuberculosis* in response to Kanamycin have been observed (Habib et al., 2017).

The use of aminoglycosides in the clinic has led to the development of resistance. Aminoglycoside resistance occurs commonly through modification of the aminoglycosides, methylation of their target rRNA or overexpression of efflux pumps (Nikaido, 2009). Modification of the aminoglycosides is achieved by aminoglycoside acetyltransferases (AAC) for acetylation, aminoglycoside adenyltransferase (AAD) for adenylylation, or phosphotransferases for phosphorylation (Mingeot-Leclercq et al., 1999). Aminoglycoside resistance is known to be inducible (Lovett and Rogers, 1996). One mechanism of induction of aminoglycosides resistance has been identified. The aminoglycosides can bind to riboswitch RNA in *Pseudomonas fluorescens* to regulate translation of AAC or AAD expression (He et al., 2013; Jia et al., 2013; Chen and Murchie, 2014; Wang et al., 2019; Zhang et al., 2020). The aminoglycoside sensing riboswitch is located at the 5′ untranslated region of AAC or AAD. Reporter assays in *E. coli* showed that aminoglycosides specifically induce expression of a reporter gene through the riboswitch RNA (Jia et al., 2013). Direct binding of aminoglycosides and the riboswitch RNA was measured. Chemical probing showed that aminoglycoside binding to the riboswitch RNA induces a structural change in Shine-Delgarno sequence (bacterial ribosomal binding site) that in turn regulate the translation of AAC or AAD.

These evidences indicate that the aminoglycoside has wider and diverse cellular functions as well as act as antibiotics. Here we have carried out transcriptome analysis by RNA-sequencing (RNA-seq) to investigate the genome-wide effect of Kanamycin B on *E. coli*, focusing on founding non-antibiotic cellular functions and kanamycin binding RNA. We identified the differentially expressed genes (DEGs) that are mainly related to microbial metabolism in diverse environments, two-component systems, nitrogen metabolism upon Kanamycin B treatment. We further showed that Kanamycin B could bind to 5′ UTR of the DEG and induce reporter gene expression. The results provide an insight into a better understanding of the wider aminoglycosides function in *E. coli*.

## Materials and methods

### Bacterial strains and growth condition

The strain used in this study was *E. coli* K-12 derived JM109 strain (Sangong, China). A single colony of JM109 was inoculated in 5 ml of rich medium LB and cultured overnight at 37°C under the condition of shaking speed at 200 rpm. The overnight bacterial culture was diluted 1: 1000 in fresh LB at 0, 0.5, 1, and 2 μM Kanamycin B. OD600 was measured using a SpectraMax® M5 every 1 h for 12 h. Each experiment was replicated three times.

### RNA extraction and library preparation for transcriptome sequencing

*E. coli* JM09 was cultured with 0, 0.5, and 1 μM Kanamycin B for 7 h to an OD600 ≈ 0.6. Total RNA of harvested cells was extracted using Trizol (Invitrogen, United States). Total RNA of each sample was quantified and qualified by Agilent 2100 Bioanalyzer (Agilent Technologies, Palo Alto, CA, United States), NanoDrop (Thermo Fisher Scientific Inc.), and 1% agarose gel. The NEBNext Ultra II Directional RNA Library Prep Kit was used for the preparation of the library. The library preparations were performed following the manufacturer’s protocol. Three biological replicates of each library were sequenced on an Illumina HiSeq X Ten (Illumina, San Diego, CA, United States) with 150-bp paired-ends by AZENTA (Suzhou, China). RNA-seq data were uploaded to the Sequence Read Archive of the National Center for Biotechnology Information (accession number: PRJNA756617).

### Sequence reads mapping and assembly

The raw reads of fastq format were initially filtered by removing reads containing adaptors, poly-N and low-quality reads. The high-quality clean reads were obtained (reads contain 20% base quality lower than Q20). Q20, Q30, GC-content and sequence duplication level of the clean data were calculated. The analyses were performed by using high quality clean reads. The clean reads were subsequently mapped to the *E. coli* str. K-12 substr. MG1655, genome assembly (NCBI: NC_000913.3) by using bowtie2 v2.2.6.

### Principal component analysis

Principal Component Analysis (PCA) was performed to investigate the reproducibility of the biological repeats. DESeq2 in the deseq2_qc.r script was used for PCA calculation. The scores of the first and second principal components were plotted into two-dimensional space to represent the spatial relationships within the repeat samples for visualization (Long et al., 2020).

### Differential expression analysis and functional annotations of DEGs

The expression levels of genes were counted by using HTseq in each sample (Anders et al., 2015). The DEGs are filtered to meet the threshold level with a |fold change| ≥ 2, and false discovery rate value of *p* < 0.05 in each pairwise comparison by using edgeR (Robinson et al., 2010). Functional annotations of DEGs are performed by R package, ClusterProfiler (Yu et al., 2012) with the Kyoto Encyclopedia of Genes and Genomes (KEGG) database. KEGG pathways with *p* < 0.05 were considered as significantly enriched.

### Hierarchical clustering analysis

Hierarchical clustering analysis of DEGs was carried out by using the function heatmap. Two of gplots (version 3.0.1) in R version 3.3.1. The heatmaps are the outcome of this analysis to represent the reproducibility of the biological repeats (Khan et al., 2019).

### Series-cluster analysis and functional annotation of the clusters

Short Time-series Expression Miner, version 1.3.13 (STEM) as a non-parametric clustering algorithm is designed to analyze short timeseries or concentration series expression data (Ernst and Bar-Joseph, 2006). STEM is a new clustering method that can identify real patterns from patterns. It clusters genes based on a series of pre-defined patterns (expression profiles). A profile is considered meaningful if the number of genes assigned to it surpasses the number of genes that are estimated to occur by chance with *p* < 0.05. The functional annotations of the clusters were analyzed by Gene ontology (GO) that is imbedded in the STEM. GO enrichment analysis is considered significant if the value of *p* < 0.05.

### Validation of DEGs by real-time quantitative PCR

The 9 RNA samples for transcriptome sequencing were also used to perform real-time quantitative PCR (RT-qPCR) to assess the reliability of the sequencing data. RNA were reverse transcribed to cDNA using by PrimeScript™ RT reagent Kit (Takara, Japan) by reverse transcription. Obtained cDNAs were further used for RT-qPCR. The RT-qPCR reactions were carried out on a 7,500 Real-Time PCR System (Applied Biosystems) using the TB Green® Premix Ex Taq™ II (Takara, Japan) according to the manufacturer’s instructions. For absolute quantification of DEGs copy number, a gene-specific PCR of known concentration was prepared as a standard. The primers were obtained from AZENTA (Suzhou, China; Supplementary Table S1). Melting curve analysis was utilized to verify specificity of all PCR products. Error bars are the standard deviation of three independent experiments.

### Reporter plasmid construction

The DNA sequences corresponding to the 5′ UTR of *napF* or *narK* were cloned into the reporter vector pGEX-leaderRNA-aac/aadlacZα as described before (Jia et al., 2013) to generate reporter plasmid pGEX-napF 5′ UTR-lacZα or pGEX-narK 5′ UTR-lacZα. The JM109 competent cells are placed on ice for 30 min. 1 μl of pGEX-napF 5′ UTR-lacZα plasmid was added to 50 μl of competent cells for transformation. The mixture was incubated on ice for 30 min. The cells were heat-shocked at 42°C in a heating block for 90 s followed by cooling down on ice for 2 min. Transfer the transformed competent cells onto LB plates containing Ampicillin (100 μg/ml). Spread with aseptic spreader uniformly until it gets resistant to move. Invert the plates and incubate at 37°C overnight and transformed colonies should appear in 12–16 h.

### The reporter assays: Agar diffusion and β-galactosidase assays

Five milliliters culture of JM109 cells transformed with the pGEX-napF 5′ UTR-lacZα reporter plasmid was incubated overnight in LB broth containing 100 μg/ml Ampicillin. The culture was diluted 1: 100 in fresh LB broth containing Ampicillin (100 μg/ml) and IPTG (0.5 mM) and incubated for 2 h. A total of 1 ml of cultured cells (an OD600 ≈ 0.3) was then mixed well with 10 ml of 0.6% LB agar at 45°C. After mixing of components, the cells was poured on a 1.5% LB agar plate containing 100 μg/ml Ampicillin, 0.5 mM IPTG, and 200 μg/ml X-Gal. For the control sample IPTG was not added in the culture or on the agar plate. After the agar had solidified, discs of filter paper spotted with 2 μl different antibiotics stock solutions were placed onto agar plates and incubated at 37°C for at least 18 h (Bailey et al., 2008). Stock solutions included Kanamycin B (10 mM), Sisomycin (10 mM), Neamine (150 mM), Amikacin (10 mM), Gentamycin (10 mM), Tobramycin (10 mM), Ribostamycin (10 mM), Paromomycin (10 mM), Levofloxacin (5 mM), Tetracycline (10 mM), Erythromycin (100 mM), Trimethoprim (5 mM). β-Galactosidase assays were performed as previously described (Zhang and Bremer, 1995). Error bars are the standard deviation of three independent experiments.

### MicroScale thermophoresis

The 5′ UTR of *napF* RNAs were prepared by *in vitro* transcription using T7 RNA polymerase. After PAGE purification, RNA was labeled with fluorescein-5-thiosemicarbazide as previously described (Wu et al., 1996). The fluorescein-RNA was annealed separately with 40 mM HEPES (pH 7.4), 100 mM KCl, the mixture was heated at 95°C for 3 min followed by subsequently placed on ice for 3 min. MgCl_2_ was added to a final concentration of 2.5 mM. Series of aminoglycosides were prepared with a final concentration from 125 nM to 128 μM. After incubation at 25°C for 20 min. The samples were added to premium coated capillaries (NanoTemper Technologies, GmbH, Munich, Germany) and subsequently subjected to MST analysis (20% MST power, 30% LED power) on a Monolith NT.115 pico instrument at 25°C. And data analysis was performed by MO. Affinity Analysis software (Entzian and Schubert, 2016; Moon et al., 2018). Error bars are the standard deviation of three independent experiments. The signal-to-noise ratio reflects the quality of the binding data and a ratio more than 12 reflects an excellent assay.

## Results

### Overview of the RNA-seq data

**Figure 1:**
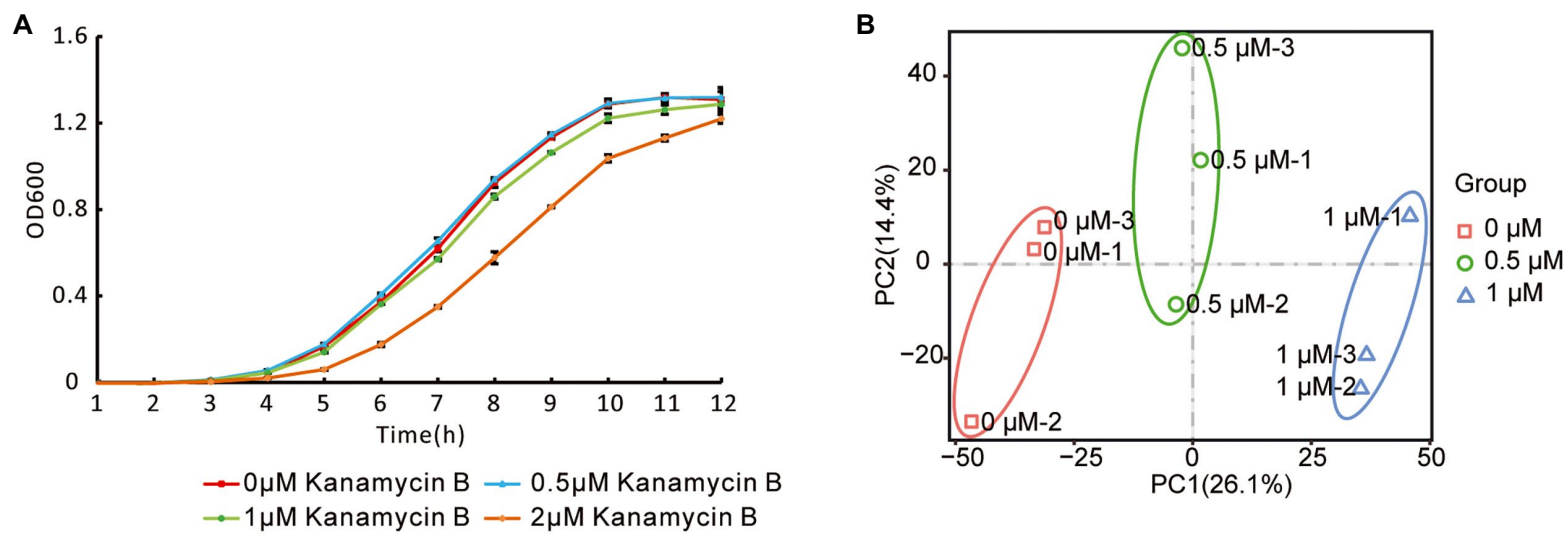
*Escherichia coli* growth curve and reproducibility of RNA-seq data. **(A)** Growth curve of JM109 treated with or without Kanamycin B. JM109 cells was grown in the presence of 0, 0.5, 1, and 2 μM Kanamycin B. Cell density was measured hourly. Error bars are the standard deviation of three independent experiments. **(B)** Principal component analysis of the RNA-Seq data. The small icon represents the original samples, colors are used to differentiate between treatments: red (0 μM), green (0.5 μM), and blue (1 μM).

To investigate the effect of Kanamycin B on *E. coli* JM109, we performed transcriptome-profiling analysis by RNA-seq in the absence or presence of Kanamycin B. Since Kanamycin B is a bactericidal antibiotic, initial cultures in the absence or presence of a broad range of Kanamycin B were cultivated to select concentrations that are neither lethal nor inhibit growth (Figure 1A). 0.5 or 1 μM of Kanamycin B showed minimal effects on cell growth, while 2 μM of Kanamycin B clearly inhibited growth. As a result, 0.5 and 1 μM of Kanamycin B were chosen for transcriptome profiling analysis. Total RNA was extracted from cells at mid-log phase that had been treated with 0, 0.5, and 1 μM of Kanamycin B for RNA-seq analysis. The transcriptome-profiling data was uploaded to the National Center for Biotechnology Information Sequence Read Archive (accession number PRJNA756617). With three biological repeats of each sample, a total of 9 cDNA libraries were constructed containing 340.20 million raw reads; 339.64 million clean reads (accounting for 99.83% of raw reads) were recorded after removing adapter sequences and reads of low quality and those with more than 5% N bases. The average number of clean reads per sample was about 37.74 million and the clean Q20 (sequencing error rate < 1%) base rate was >97.86% for each sample. Ultimately, 336.74 million high-quality reads (accounting for 99.14% of clean reads) were mapped to the *E. coli* str. K-12 substr. MG1655 genome.

To investigate their reproducibility, the biological repeats were analyzed by PCA. The results revealed that PC1 and PC2 had values of 26.1% and 14.4%, respectively, and accounted for 40.5% of the principal components (Figure 1B). PCA showed consistency between the three replicates at each Kanamycin B concentration; samples from the biological repeat clustered together, reflecting minimal differences between them. PCA suggested that the three biological repeats in this study were reasonably reproducible (Figure 1B).

### Gene expression profile in response to Kanamycin B treatment

**Figure 2:**
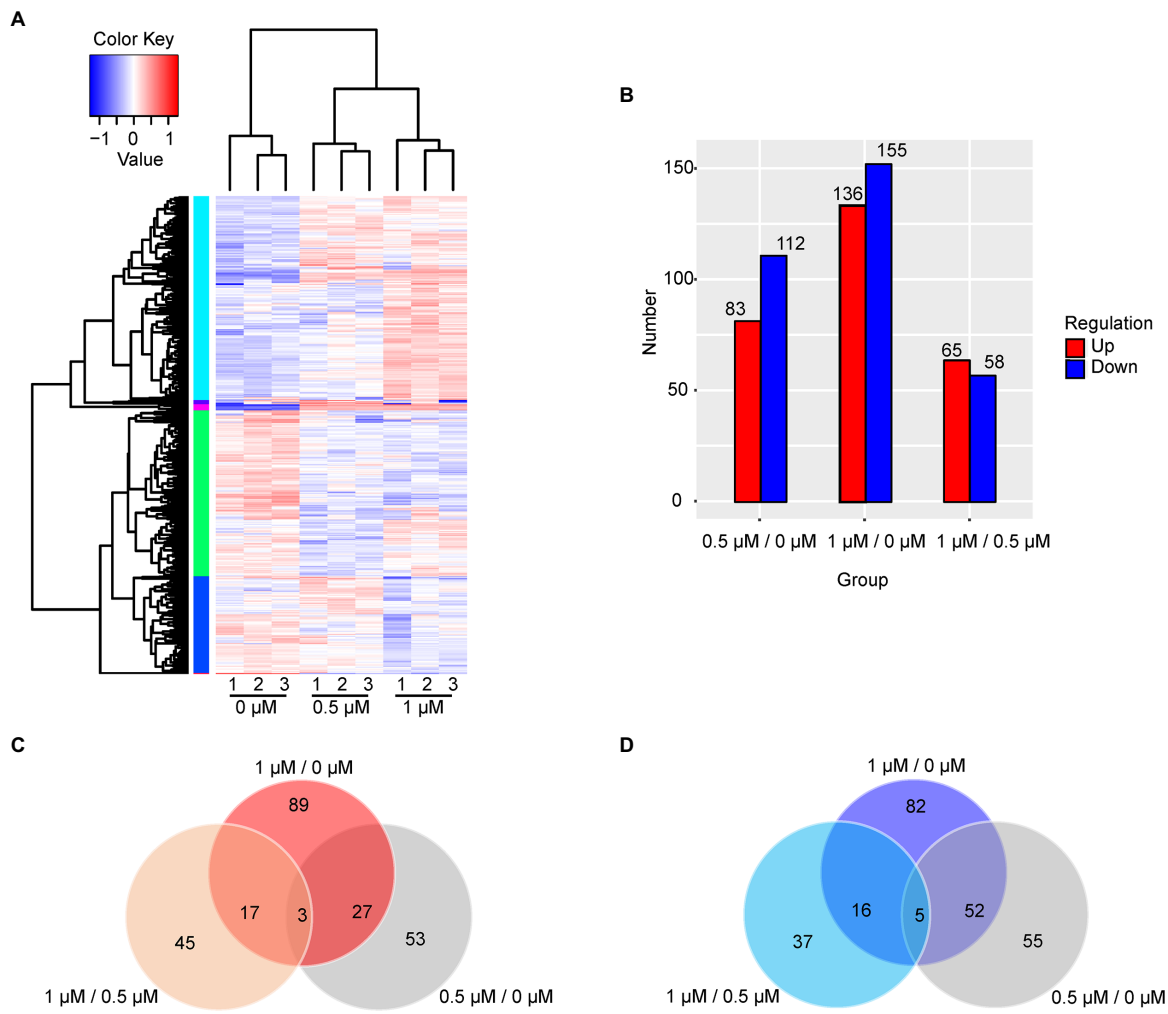
Analysis of DEGs. **(A)** The hierarchical cluster analysis of 463 DEGs. Rows represent gene expression levels and columns represent concentration of Kanamycin B, treatment and repeats. The relative quantitative changes within the row are shown in color: red suggests a relative higher expression whereas blue suggests a relative lower expression level. **(B)** The number of DEGs in the comparison groups is presented in histogram. Upregulated DEGs (red), and downregulated DEGs (blue) were presented by histogram. **(C)** The Venn diagram displays the number of the shared and unique genes of upregulated DEGs. **(D)** The Venn diagram displays the number of the shared and unique genes of downregulated DEGs.

The cellular response to Kanamycin B was revealed by the changes in the levels of gene expression. The DEGs in response to treatment by Kanamycin B were identified by selecting gene expression levels with |fold changes| ≥ 2 and significant differences in *p*-values of <0.05 at 0.5 or 1 μM. Figure 2A shows a cluster analysis of the DEGs (for a full list of DEGs; see Supplementary Table S2). There are two main clusters: one with genes that were induced and one with genes that were repressed in response to Kanamycin B treatment. We identified 83 or 136 induced DEGs and 112 or 155 repressed DEGs upon treatment with 0.5 or 1 μM of Kanamycin B, respectively, compared to no treatment (Figure 2B). There are 65 upregulated and 58 downregulated DEGs in the 1/0.5 μM (comparisons between sample treated with 1 and 0.5 μM Kanamycin B) group (Figure 2B). Fewer numbers of DEGs were observed in the 1/0.5 μM group compared to the 0.5/0 μM and 1/0 μM (comparisons between sample treated with 0.5 or 1 μM Kanamycin B and the control) groups suggesting that 0.5 or 1 μM Kanamycin B causes similar cellular responses. We next compared the similarities and differences of the DEGs in response to 0.5 or 1 μM Kanamycin B treatment. The numbers of overlapping genes between the groups (0.5/0 μM, 1/0 μM, and 1/0.5 μM) are shown in the Venn diagram for upregulated or downregulated DEGs (Figures 2C,D) and overlapping groups listed (Supplementary Table S3). There is considerable overlap between 0.5/0 μM and 1/0 μM DEG groups; 30 genes and 57 genes were co-induced or co-repress in 0.5 and 1 μM of Kanamycin B treatment. All of the DEGs represent 8.8% of the whole genome transcripts. In addition, the results show that the magnitude of the transcriptional responses in DEGs varied significantly from gene to gene. For example, some genes are upregulated over 10-fold upon Kanamycin B treatment while the expression of many other genes are induced only by about 2-fold (Table 1; Supplementary Table S2).

**Table 1:** Functional classification of DEGs (1/0 μM).

Gene symbol	Fold change	Regulation	Location	Gene annotation
**Microbial metabolism in diverse environments**				
nirD*	219.45	Up	Cytosol	Nitrite reductase subunit NirD
*nirB*	5.38	Up	Cytosol	Nitrite reductase catalytic subunit NirB
*narI*	11.30	Up	Inner membrane	Nitrate reductase A subunit γ
*narG*	9.58	Up	Inner membrane	Nitrate reductase A subunit α
*narH*	7.48	Up	Inner membrane	Nitrate reductase A subunit β
*ydhX*	3.61	Up	Periplasmic space	Putative 4Fe-4S ferredoxin-like protein YdhX
*glcD*	3.09	Up	Cytosol	Glycolate dehydrogenase, putative FAD-linked subunit
*glpE*	2.75	Up	Cytosol	Thiosulfate sulfurtransferase GlpE
*yqeF*	2.39	Up	Cytosol	Putative acyltransferase
*lysC*	2.14	Up	Cytosol	Aspartate kinase III
*frdD*	−9.37	Down	Inner membrane	Fumarate reductase membrane protein FrdD
*frdB*	−9.33	Down	Inner membrane, cytosol	Fumarate reductase iron–sulfur protein
*frdA*	−6.35	Down	Inner membrane, cytosol	Fumarate reductase flavoprotein subunit
*frdC*	−3.00	Down	Inner membrane	Fumarate reductase membrane protein FrdC
*hyaB*	−4.88	Down	Periplasmic space, inner membrane	Hydrogenase 1 large subunit
*hyaA*	−3.41	Down	Inner membrane	Hydrogenase 1 small subunit
*adhE*	−2.86	Down	Cytosol	Alcohol dehydrogenase/aldehyde-dehydrogenase
*adhP*	−2.67	Down	Cytosol	Ethanol dehydrogenase / alcohol dehydrogenase
*gabT*	−3.19	Down	Cytosol	4-aminobutyrate aminotransferase GabT
*gabD*	−2.68	Down	Cytosol	Succinate-semialdehyde dehydrogenase (NADP+) GabD
*gadA*	−2.43	Down	Cytosol	Glutamate decarboxylase A
*gadB*	−2.10	Down	Cytosol, membrane	Glutamate decarboxylase B
*hcaE*	−2.86	Down	No annotation	Putative 3-phenylpropionate/cinnamate dioxygenase subunit α
*paaK*	−2.73	Down	Cytosol	Phenylacetate-coa ligase
*yghX*	−2.57	Down	No annotation	Putative hydrolase fragment
*aldB*	−2.43	Down	Cytosol	Aldehyde dehydrogenase B
*fbaB*	−2.19	Down	Cytosol	Fructose-bisphosphate aldolase class I
*tktB*	−2.19	Down	Cytosol	Transketolase 2
*allB*	−2.16	Down	Cytosol	Allantoinase
**Two-component system**				
*fdnG*	19.98	Up	Periplasmic space	Formate dehydrogenase n subunit α
*fdnH*	11.67	Up	Inner membrane	Formate dehydrogenase n subunit β
*fdnI*	5.38	Up	Inner membrane	Formate dehydrogenase n subunit γ
*narI*	11.30	Up	Inner membrane	Nitrate reductase a subunit γ
*narG*	9.58	Up	Inner membrane	Nitrate reductase a subunit α
*narH*	7.48	Up	Inner membrane	Nitrate reductase a subunit β
*narJ*	6.84	Up	Cytosol	Nitrate reductase 1 molybdenum cofactor assembly chaperone
*narX*	2.24	Up	Periplasmic space, inner membrane	Sensory histidine kinase NarX
*rstB*	2.50	Up	Inner membrane	Sensory histidine kinase RstB
*uhpB*	2.16	Up	Inner membrane	Sensory histidine kinase UhpB
*baeS*	2.20	Up	Inner membrane	Sensor histidine kinase BaeS
*rcsA*	3.35	Up	Cytosol	DNA-binding transcriptional activator RcsA
*ompF*	2.98	Up	Outer membrane	Outer membrane porin F
*yqeF*	2.39	Up	Cytosol	Putative acyltransferase
*uhpT*	2.19	Up	Inner membrane	Hexose-6-phosphate:phosphate antiporter
*frdD*	−9.37	Down	Inner membrane	Fumarate reductase membrane protein FrdD
*frdB*	−9.33	Down	Inner membrane, cytosol	Fumarate reductase iron–sulfur protein
*frdA*	−6.35	Down	Inner membrane, cytosol	Fumarate reductase flavoprotein subunit
*frdC*	−3.00	Down	Inner membrane	Fumarate reductase membrane protein FrdC
*appB*	−6.19	Down	Inner membrane	Cytochrome bd-ii ubiquinol oxidase subunit ii
*appC*	−4.33	Down	Inner membrane	Cytochrome bd-ii ubiquinol oxidase subunit i
*hyaC*	−7.15	Down	Inner membrane	Hydrogenase 1 cytochromebsubunit
*cusA*	−2.99	Down	Inner membrane	Copper/silver export system rnd permease
*mdtC*	−2.58	Down	Inner membrane	Multidrug efflux pump rnd permease subunit MdtC
**Butanoate metabolism**				
*yqeF*	2.39	Up	Cytosol	Putative acyltransferase
*frdD*	−9.37	Down	Inner membrane	Fumarate reductase membrane protein FrdD
*frdB*	−9.33	Down	Inner membrane, cytosol	Fumarate reductase iron–sulfur protein
*frdA*	−6.35	Down	Inner membrane, cytosol	Fumarate reductase flavoprotein subunit
*frdC*	−3.00	Down	Inner membrane	Fumarate reductase membrane protein FrdC
*gabT*	−3.19	Down	Cytosol	4-aminobutyrate aminotransferase GabT
*gabD*	−2.68	Down	Cytosol	Succinate-semialdehyde dehydrogenase (NADP+) GabD
*gadA*	−2.43	Down	Cytosol	Glutamate decarboxylase a
*gadB*	−2.10	Down	Cytosol, membrane	Glutamate decarboxylase b
*adhE*	−2.86	Down	Cytosol	Alcohol dehydrogenase/aldehyde-dehydrogenase
*dmlA*	−2.01	Down	Cytosol	D-malate/3-isopropylmalate dehydrogenase (decarboxylating)
**Nitrogen metabolism**				
nirD*	219.45	Up	Cytosol	Nitrite reductase subunit NirD
*nirB*	5.38	Up	Cytosol	Nitrite reductase catalytic subunit NirB
*narK*	16.98	Up	Inner membrane	Nitrate:nitrite antiporter NarK
*narI*	11.30	Up	Inner membrane	Nitrate reductase a subunit γ
*narG*	9.58	Up	Inner membrane	Nitrate reductase a subunit α
*narH*	7.48	Up	Inner membrane	Nitrate reductase a subunit β
*hcp*	4.03	Up	Cytosol	Proteins-nitrosylase
*can*	2.41	Up	Cytosol	Carbonic anhydrase 2
**Oxidative phosphorylation**				
*cyoA*	2.48	Up	Inner membrane	Cytochromebo3ubiquinol oxidase subunit 2
*ppa*	2.11	Up	Cytosol	Inorganic pyrophosphatase
*frdD*	−9.37	Down	Inner membrane	Fumarate reductase membrane protein FrdD
*frdB*	−9.33	Down	Inner membrane, cytosol	Fumarate reductase iron–sulfur protein
*frdA*	−6.35	Down	Inner membrane, cytosol	Fumarate reductase flavoprotein subunit
*frdC*	−3.00	Down	Inner membrane	Fumarate reductase membrane protein FrdC
*appB*	−6.19	Down	Inner membrane	Cytochrome bd-ii ubiquinol oxidase subunit ii
*appC*	−4.33	Down	Inner membrane	Cytochrome bd-ii ubiquinol oxidase subunit i
**Alanine, aspartate, and glutamate metabolism**				
*gabT*	−3.19	Down	Cytosol	4-aminobutyrate aminotransferase GabT
*gabD*	−2.68	Down	Cytosol	Succinate-semialdehyde dehydrogenase (NADP+) GabD
*gadA*	−2.43	Down	Cytosol	Glutamate decarboxylase a
*gadB*	−2.10	Down	Cytosol, membrane	Glutamate decarboxylase b
*aspA*	−2.49	Down	Cytosol	Aspartate ammonia-lyase
*glsA*	−2.40	Down	No annotation	Glutaminase 1
**Sulfur metabolism**				
*ydhX*	3.61	Up	Periplasmic space	Putative 4fe-4s ferredoxin-like protein YdhX
*glpE*	2.75	Up	Cytosol	Thiosulfate sulfurtransferase GlpE
*sbp*	2.05	Up	Periplasmic space	Sulfate/thiosulfate abc transporter periplasmic binding protein Sbp
*dmsB*	−4.23	Down	Inner membrane	Dimethyl sulfoxide reductase subunit b
*dmsC*	−3.93	Down	Inner membrane	Dimethyl sulfoxide reductase subunit c
*dmsA*	−2.49	Down	Inner membrane	Dimethyl sulfoxide reductase subunit a
**Arginine and proline metabolism**				
*puuD*	3.81	Up	Cytosol	γ-glutamyl-γ-aminobutyrate hydrolase
*puuC*	3.22	Up	Cytosol	γ-glutamyl-γ-aminobutyraldehyde dehydrogenase
*puuA*	2.96	Up	Cytosol	Glutamate-putrescine ligase
*speG*	2.31	Up	Cytosol	spermidine N-acetyltransferase
*patA*	−2.16	Down	Cytosol	Putrescine aminotransferase
**beta-alanine metabolism**				
*gabT*	−3.19	Down	Cytosol	4-aminobutyrate aminotransferase GabT
*gabD*	−2.68	Down	Cytosol	Succinate-semialdehyde dehydrogenase (NADP+) GabD
*gadA*	−2.43	Down	Cytosol	Glutamate decarboxylase a
*gadB*	−2.10	Down	Cytosol, membrane	Glutamate decarboxylase b
**Selenocompound metabolism**				
*ynfE*	−9.15	Down	Periplasmic space, inner membrane	Putative selenate reductase YnfE
*ynfF*	−5.26	Down	Periplasmic space, inner membrane	Putative selenate reductase YnfF
*sufS*	−2.31	Down	Cytosol	L-cysteine desulfurase
metE	−2.15	Down	Cytosol	Cobalamin-independent homocysteine transmethylase
**Tyrosine metabolism**				
*adhE*	−2.86	Down	Cytosol	Alcohol dehydrogenase/aldehyde-dehydrogenase
*adhP*	−2.67	Down	Cytosol	Ethanol dehydrogenase / alcohol dehydrogenase
gabD	−2.68	Down	Cytosol	Succinate-semialdehyde dehydrogenase (NADP+) GabD
**Chloroalkane and chloroalkene degradation**				
*adhE*	−2.86	Down	Cytosol	Alcohol dehydrogenase/aldehyde-dehydrogenase
*adhP*	−2.67	Down	Cytosol	Ethanol dehydrogenase/alcohol dehydrogenase
**Naphthalene degradation**				
*adhE*	−2.86	Down	Cytosol	Alcohol dehydrogenase/aldehyde-dehydrogenase
*adhP*	−2.67	Down	Cytosol	Ethanol dehydrogenase/alcohol dehydrogenase
**Nitrotoluene degradation**				
*hyaB*	−4.88	Down	Periplasmic space, inner membrane	Hydrogenase 1 large subunit
*hyaA*	−3.41	Down	Inner membrane	Hydrogenase 1 small subunit
**Polyketide sugar unit biosynthesis**				
*rfbD*	2.97	Up	Cytosol	Dtdp-4-dehydrorhamnose reductase
*rfbA*	2.48	Up	Cytosol	Dtdp-glucose pyrophosphorylase
**Taurine and hypotaurine metabolism**				
*gadA*	−2.43	Down	Cytosol	Glutamate decarboxylase a
**Chlorocyclohexane and chlorobenzene degradation**				
*yghX*	−2.57	Down	No annotation	Putative hydrolase fragment
*gadB*	−2.10	Down	Cytosol, membrane	Glutamate decarboxylase b
Fluorobenzoate degradation				
*yghX*	−2.57	Down	No annotation	Putative hydrolase fragment
Inositol phosphate metabolism				
*appA*	−3.96	Down	Periplasmic space	Periplasmic phosphoanhydride phosphatase/multiple inositol-polyphosphate phosphatase
**Toluene degradation**				
*yghX*	−2.57	Down	No annotation	Putative hydrolase fragment
**Biosynthesis of ansamycins**				
*tktB*	−2.19	Down	Cytosol	Transketolase 2
**Synthesis and degradation of ketone bodies**				
*yqeF*	2.39	Up	Cytosol	Putative acyltransferase

* The counts of the *nirD* gene is 0.

### Functional classification of DEGs

**Figure 3:**
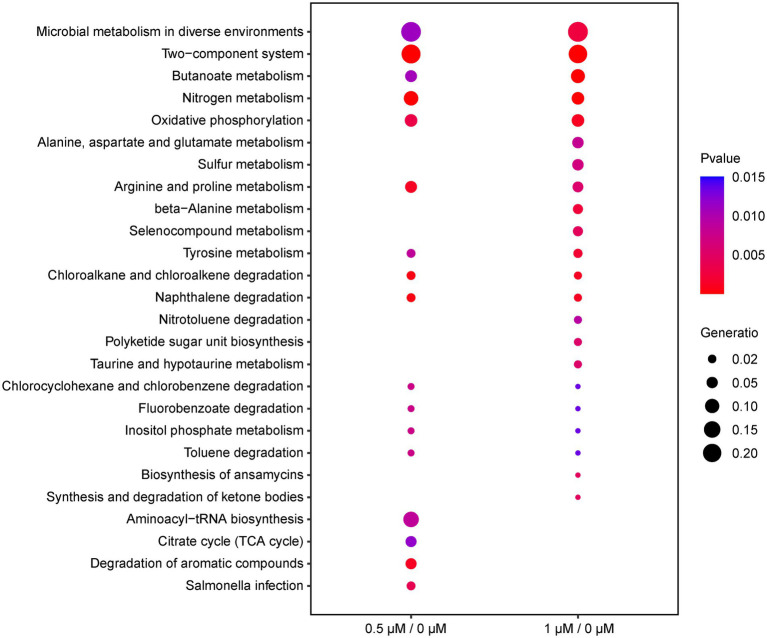
Functional classification of DEGs (0.5/0 μM and 1/0 μM) by KEGG enrichment analysis under the cutoff of *p*-value < 0.05. The ordinate represents the name of the pathway; the diameter of the dots implies the gene ratio, ranging from 0.02 to 0.20 (smallest to biggest dot); the color of the plot corresponds to *p*-value.

**Figure 4:**
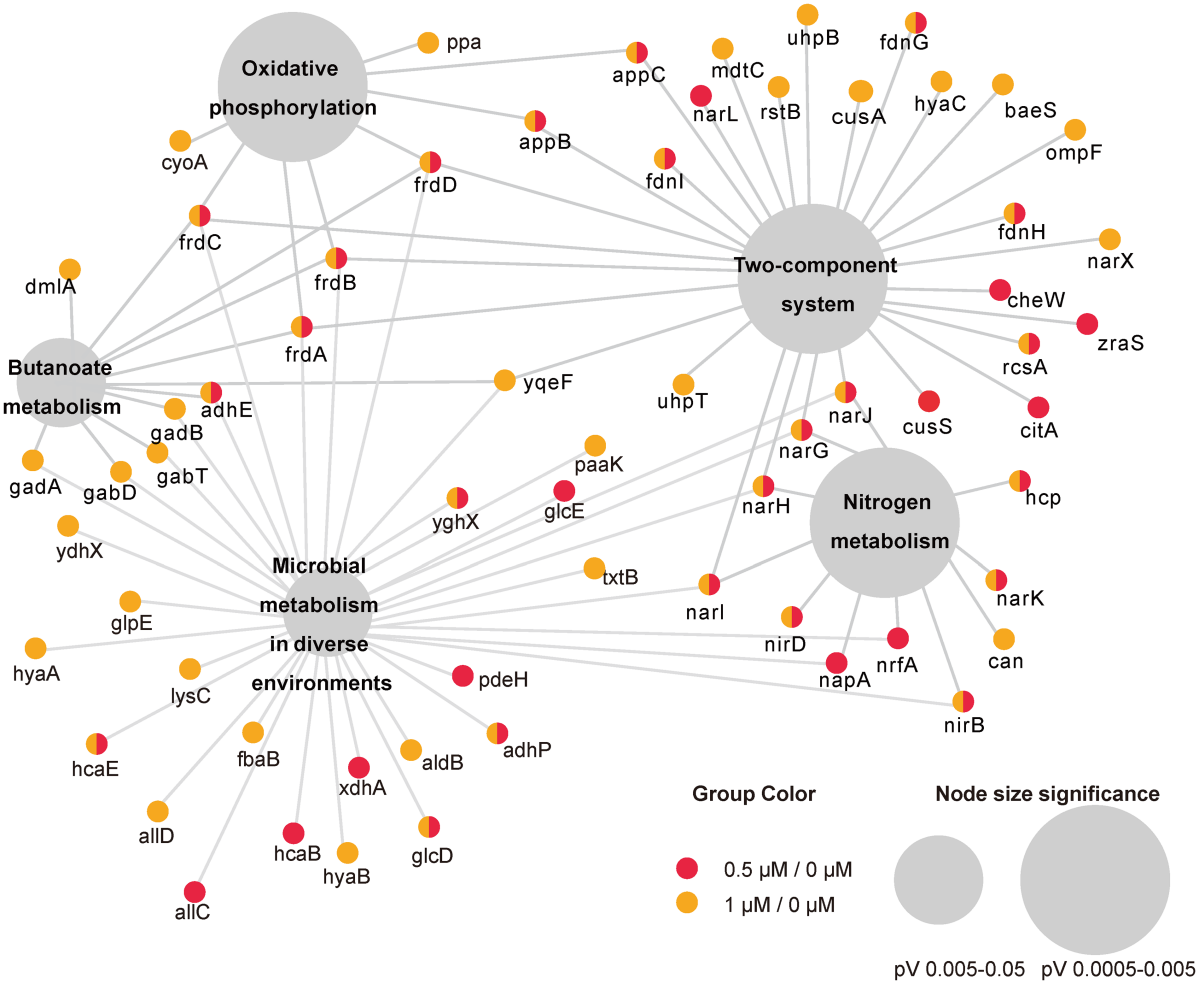
Network representation of the five main enriched KEGG pathways under the cutoff of *p*-value < 0.05 and their associated DEGs in two comparisons (0.5/0 μM and 1/0 μM). Small circles represent genes involved whereas colors represent DEGs in different comparison. The large gray circles are the KEGG pathway, and the size of the circles reflect the value of *p*.

To investigate the function of the DEGs, we carried out a KEGG pathway analysis of DEGs. Table 1 (1/0 μM), Supplementary Table S4 (0.5/0 μM), and Figure 3 show the DEGs classified into known or predicted functional groups. These genes encode proteins involved in the following cellular functions: microbial metabolism in diverse environments, two-component systems, nitrogen metabolism, butanoate metabolism, arginine and proline metabolism and inositol phosphate metabolism. Genes that encode proteins that participate in cellular degradation, including chloroalkane and chloroalkene degradation, naphthalene degradation, chlorocyclohexane and chlorobenzene degradation, fluorobenzoate degradation, toluene degradation and degradation of aromatic compounds. Genes that encode proteins involved in oxidative phosphorylation, aminoacyl-tRNA biosynthesis or the TCA cycle. KEGG pathway analysis of DEGs showed that many genes are classified in more than one functional group (Table 1). Further analysis of the genes that belong to the five relatively large functional classes (two-component systems, oxidative phosphorylation, nitrogen metabolism, microbial metabolism in diverse environments and butanoate metabolism) revealed the linkage network of these DEGs (Figure 4; Table 1), suggesting an overlap between these main functional classes through these DEGs.

### Series-cluster analysis and functional annotation of the clusters

**Figure 5:**
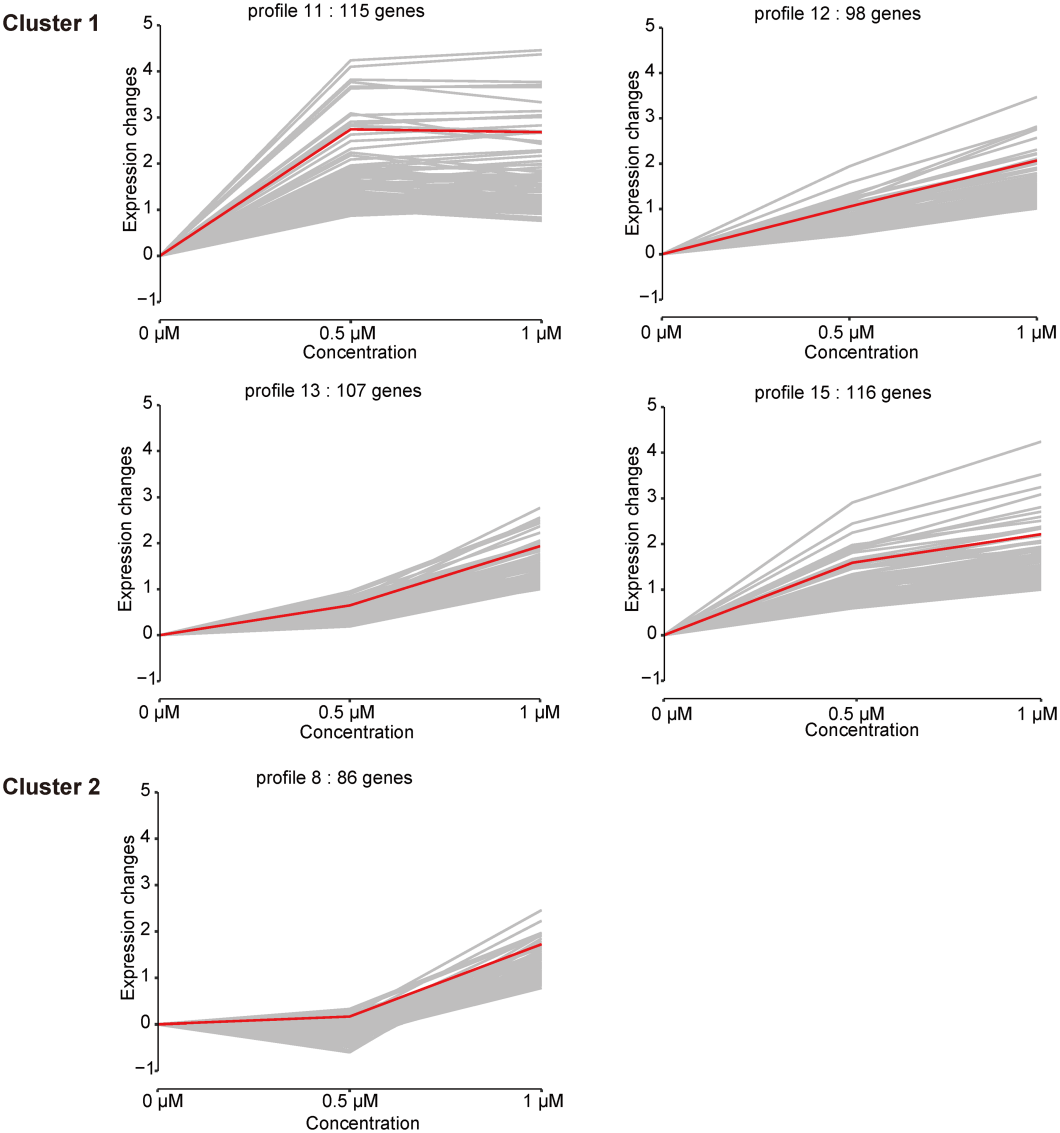
Cluster analysis of the gene expression patterns in the presence of 0, 0.5, 1 μM of Kanamycin B. Based on the correlation coefficient the genes were assigned to the model profile that more closely matched its series. Five Profiles were significantly clustered (*p* < 0.05). The number of genes in each of the clusters and each profile is shown; gray line is the trend of individual gene expression; red line is the overall trend of gene expression.

In addition to the DEGs analysis of the cellular response to Kanamycin B, we observed diverse and complex gene expression patterns over the Kanamycin B concentration courses. To dissect the gene expression patterns and further understand the gene functions, we performed a series-cluster analysis and functional annotation of the clusters. The series-cluster analysis can group genes that have similar expression patterns to form clusters of profiles and GO enrichment analysis further reveals functional annotations. A total of 4,534 genes were partitioned into 16 profiles of which 5 profiles were statistically significant (*p* < 0.05; Figure 5; Supplementary Table S5). STEM calculated the five profiles into two clusters. Each gene cluster exhibited a distinctive expression pattern. Cluster 1 includes profile 11 (115 genes), 12 (98 genes), 13 (107 genes), and 15 (116 genes). Genes in profile 11 show that expression levels increase rapidly after 0.5 μM Kanamycin B treatment and reach a plateau with 1 μM Kanamycin B, while the genes in profile 12, 13, and 15 present a gradual upregulation pattern. Cluster 2 consists of only profile 8 with 86 genes that exhibit similar expression from 0 to 0.5 μM Kanamycin B treatment and an increase in transcription with 1 μM Kanamycin B. STEM also supports GO enrichment analysis for each cluster and profile and includes functional classification of genes in the profiles in Figure 5 (Supplementary Table S6). Profile 11 includes 115 genes of which 18 GO terms are associated with nitrogen compound metabolism and oxidation–reduction processes. The profile 12 cluster contains a number of membrane genes, and the bacterial outer membrane contributes to drug efflux resistance mechanisms. The profile 13 includes 107 genes enriched in 68 GO terms that are linked to DNA binding and the regulation of RNA biosynthesis. Profile 15 has 116 genes enriched in 10 GO Terms of which 8 are related to biofilm formation and cell adhesion. Profile 8 in cluster 2 is associated with genes involved in RNA biosynthetic processes, regulation of primary metabolic process and cellular biosynthetic process.

### Validation by RT-qPCR

**Figure 6:**
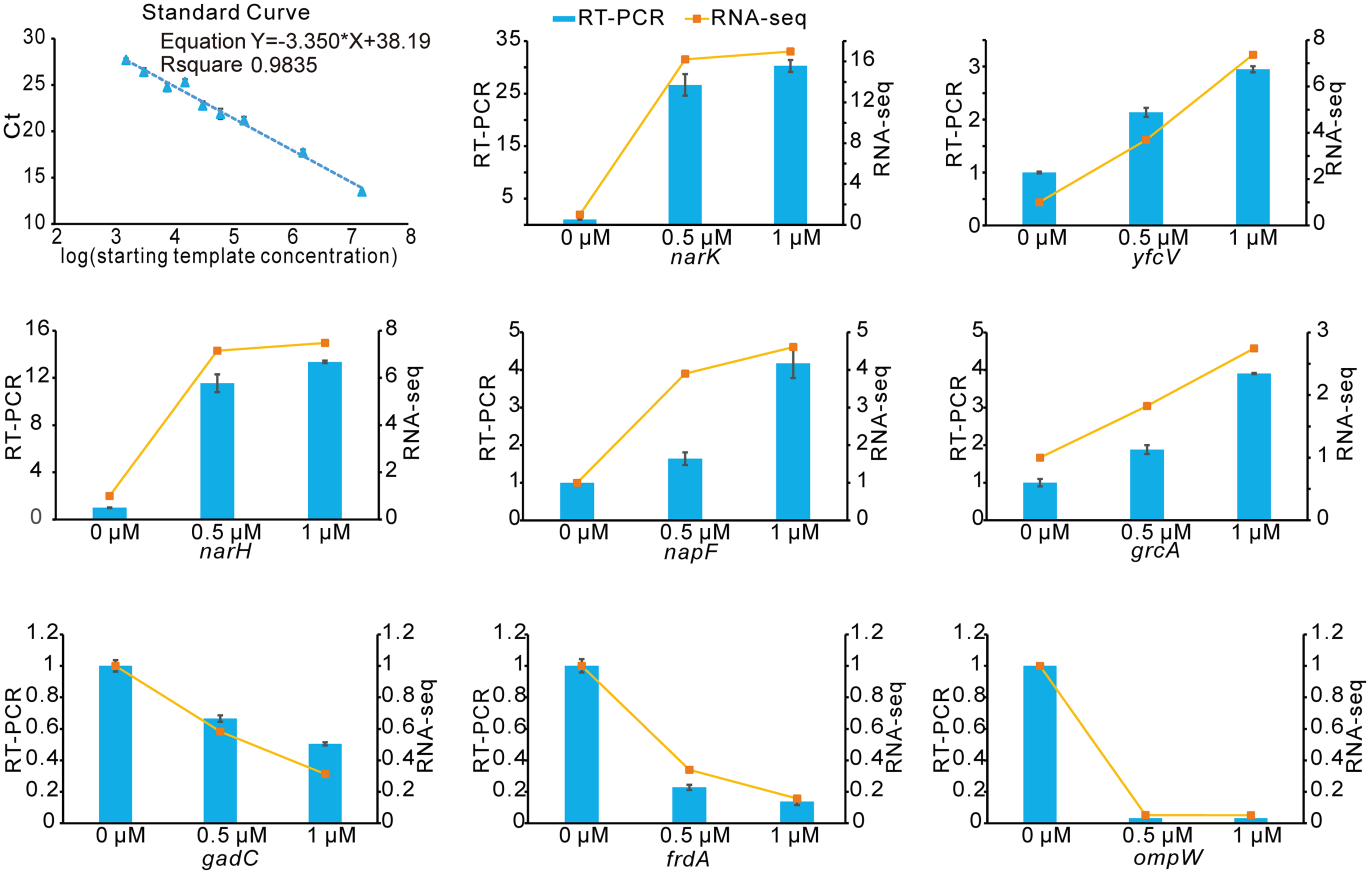
RNA-seq data validation by RT-qPCR. The blue histograms show the expression levels of 8 DEGs treated with 0.5 μM, 1 μM of Kanamycin B. The orange line indicates changes of gene expression levels upon treatment with different concentrations of Kanamycin B. Experiments were performed with three biological replicates. The right Y-axis indicates the fold change of RNA-seq data and the left Y-axis indicates the relative expression levels calculated by RT-qPCR. Error bars are the standard deviation of three independent experiments.

To verify the accuracy and reproducibility of the transcriptome results by an alternative method, we performed quantitation by RT-qPCR. We selected 8 genes that were differentially expressed upon treatment with Kanamycin B. A comparative analysis of all the selected genes showed a similar expression pattern in the RT-qPCR analysis as observed in RNAseq data (Figure 6). Thus, the RT-qPCR experiments confirm the reliability of the transcriptome sequencing data.

### The Kanamycin B and other aminoglycosides induce reporter gene expression through the UTR of the identified DEG

**Figure 7:**
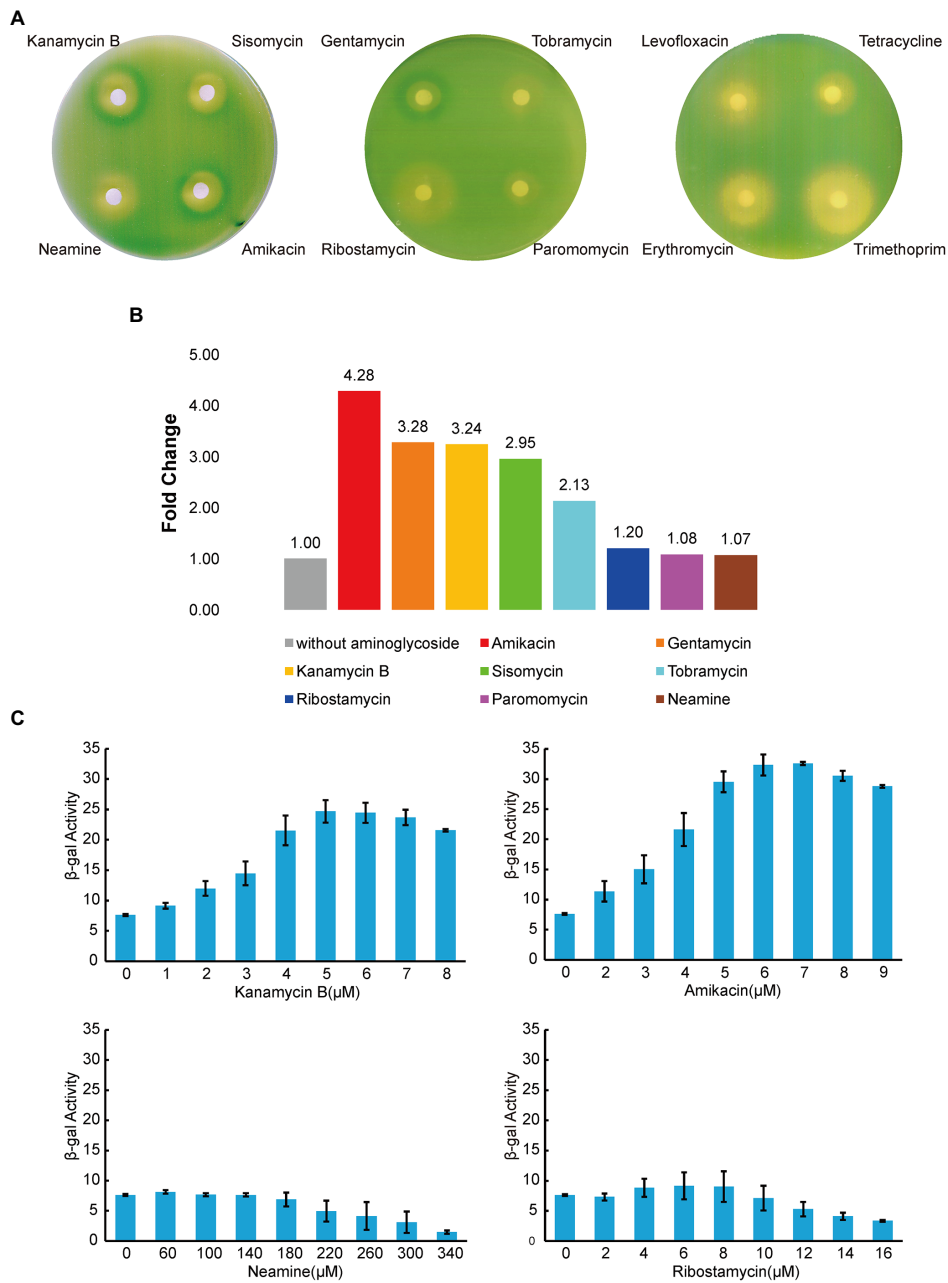
Reporter gene expression mediated by the 5′ UTR of *napF*. **(A)** Agar diffusion assay of *E. coli* JM109 transformed with the reporter construct plasmid with antibiotics in the presence of IPTG. **(B)** The induction of reporter expression in the presence of aminoglycoside antibiotics. **(C)** The β-gal activity (Miller units) of the reporter gene on titration of aminoglycoside antibiotics. Error bars are the standard deviation of three independent experiments.

The *napF* gene is one of the identified DEGs (Figure 6; Table 1) and the transcription start site of the *napF* gene has been mapped (Stewart et al., 2003). There are 80 nucleotides (nt) upstream of the transcription start site to the coding sequence of the *napF* gene. It has been shown that aminoglycosides can bind to the 5′ UTR of aac/aad gene to regulate downstream gene expression (Jia et al., 2013). To investigate whether Kanamycin B and other aminoglycoside have effects on downstream gene expression through the 5′ UTR of the *napF* gene, we constructed reporter plasmids pGEX-napF 5′ UTR-lacZα in which the 80 nt UTR of the *napF* gene and 9 nt into the coding sequence was under the control of the IPTG-inducible tac promoter (Ptac) and placed upstream of a β-galactosidase (β-gal) reporter gene. The reporter plasmid was transformed into *E. coli* JM109 and β-gal activity was tested in the absence and presence of Kanamycin B or other aminoglycoside antibiotics (Sisomycin, Tobramycin, Gentamycin, Amikacin) or control molecules (Ribostamycin, Neamine or Paromomycin and Levofloxacin, Tetracycline, Erythromycin, Trimethoprim) by agar diffusion assays. We observed induction of the reporter gene expression with Kanamycin B and Sisomycin, Amikacin, Gentamycin, and Tobramycin but not for the control molecules Ribostamycin, Neamine, Paromomycin, Levofloxacin, Tetracycline, Erythromycin or Trimethoprim (Figure 7A). No induction by Kanamycin B was seen in cells transformed with the reporter plasmid without IPTG in which Ptac promoter is inactive and the 5′ UTR of the *napF* gene is not made (Supplementary Figure S1A). No induction was observed on plates without Kanamycin B (Supplementary Figure S1B). No induction by Kanamycin B was seen in cells transformed with the reporter plasmid containing a control sequence with IPTG (Wang et al., 2019). To further quantify the agar diffusion assay, we measured β-gal activity in solution (Zhang and Bremer, 1995) on titration of Kanamycin B and other aminoglycosides (Figures 7B,C; Supplementary Figure S1C). Kanamycin B, Amikacin and Gentamycin induce the reporter gene expression by more than 3-fold. These results suggest that Kanamycin B and other aminoglycosides can induce the reporter gene expression through the 5′ UTR of the *napF* gene. The reporter gene expression requires both Kanamycin B or other aminoglycosides and the 5′ UTR of the *napF* gene. In parallel, we also observed that Kanamycin B and other aminoglycosides can induce the reporter gene expression through the 5′ UTR of the *narK* gene (another DEG; Supplementary Figure S2).

### Kanamycin B binds to 5′ UTR RNA

**Figure 8:**
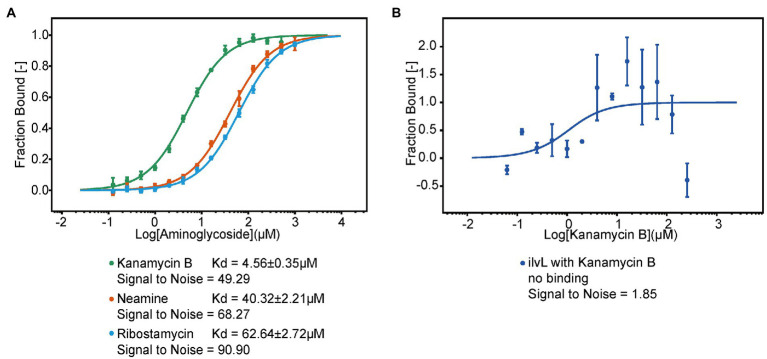
The MST binding of the 5′ UTR of *napF* with Kanamycin B and other aminoglycosides. **(A)** Binding curve generated by MST for binding of aminoglycosides to the 5′ UTR of *napF*. Error bars are standard deviations of at least three independent experiments. **(B)** Binding curve generated by MST for binding of Kanamycin B to the control RNA *ilvL*. Error bars are the standard deviation of three independent experiments.

To investigate if Kanamycin B directly bind to the 5′ UTR of the *napF* gene to induce reporter gene expression, we used MST to measure the binding of Kanamycin B or control molecule (Ribostamycin or Neamine) to the 5′ UTR of the *napF* gene or Kanamycin B to a random RNA. The 5′ UTR of the *napF* gene was prepared by *in vitro* transcription using in-house purified T7 RNA polymerase and was labeled with fluorescein-5-thiosemicarbazide as previously described (Wu et al., 1996). Binding measurements was made on a Monolith NT.115 system by NanoTemper Technologies (Entzian and Schubert, 2016; Moon et al., 2018). An increase in the measured response upon titration of the Kanamycin B was observed, indicating the formation of a Kanamycin B-RNA complex. Kanamycin B binds to the RNA with affinity at 4.5 μM. In contrast, Ribostamycin, Neamine have weaker binding and lower affinities (Figure 8A) and no binding between Kanamycin B and the control RNA (*ilvL*) was detected (Figure 8B). Thus, Kanamycin B that induces reporter gene expression can bind to the 5′ UTR of the *napF* gene.

## Discussion

Transcriptome analysis of *E. coli* JM109 treated with Kanamycin B uncovered gene expression networks that respond to Kanamycin B. We set out to investigate the extra non ribosomal inhibitory cellular (i.e., the “off target”) function of Kanamycin B in *E. coli* through transcriptome data analysis (Figures 1–6). DEGs and STEM analysis of transcriptome data revealed that Kanamycin B treatment effects the expression of genes that are involved in a variety of cellular functions including oxidative phosphorylation, nitrogen metabolism, microbial metabolism in diverse environments, biofilm formation and cell adhesion (Table 1; Figures 3–5). Kanamycin B binds to the 5′ UTR of the *napF* or *narK* gene (one of the DEGs) and induces reporter gene expression (Figure 8; Supplementary Figure S3). The results provide insights into the cellular effects of Kanamycin B and are useful for establishing the non-antibiotic function of Kanamycin B.

We compare and contrast our study with the genome-wide transcriptome analysis of *E. coli* in response to gentamycin (Zhou et al., 2019). There are several differences between the two studies in terms of growth conditions, methods of treatment and concentration of aminoglycosides. *E. coli* cell was pretreated in the presence of 1 μg/ml gentamycin (1/2 MIC) for 1 h in MHB growth medium before harvesting for RNA extraction and RNA-seq. In comparison, 0.5 μM (1/8 MIC) or 1 μM (1/4 MIC) of Kanamycin B was used in our study to treat *E. coli* cell for 7 h in LB medium prior to transcriptome analysis. These differences would expect to result in different DEGs in the two studies. However, further analysis showed some complementary findings. A high number of genes related to membrane protein and transporter functions were strongly regulated in response to gentamycin treatment. Similarly, our data (Supplementary Table S2) also showed that a considerate number of transmembrane transport genes were differentially expressed. These data from two independent studies suggest that membrane protein and transporter may have key roles in response to aminoglycosides in *E. coli*. In particular, *narK* is membrane protein and we showed biochemically that Kanamycin B may effect gene expression through binding to the 5′ UTR of *narK* (Supplementary Figure S3). In addition, both studies have identified DEGs that are involved in biological process including ribosome and translation, TCA cycle, glycolysis or carbohydrate metabolism (Supplementary Tables S2, S6; Zhou et al., 2019).

Genome-wide transcriptome profiling of *Mycobacterium tuberculosis* upon treatment with Kanamycin has been reported (Habib et al., 2017). Comparison with the functional classification and pathways of the DEGs identified in the *M. tuberculosis* study with those identified in this study in *E. coli* indicated more differences than similarities between the two studies. Notably, the diverse responses are probably due to the fundamental differences in the biology between *E. coli* and *M. tuberculosis.* However, other factors may also account for some of the different responses. The study in *M. tuberculosis* used Kanamycin instead of Kanamycin B which necessitates different growth conditions in comparison with our study.

It has been reported that the uptake of aminoglycosides into bacterial cells needs the proton motive force that is produced by electron flow through the respiratory chain of oxidative phosphorylation. The proton motive force is mainly generated by the respiratory complex I that contains membrane proteins and/or Fe-S clusters and oxidoreductases (Ezraty et al., 2013). The mechanism of aminoglycoside uptake and the bacterial cell response to the aminoglycosides is still unclear. Aminoglycoside binding riboswitches have been characterized (Jia et al., 2013) and a randomly selected Kanamycin B riboswitch has been reported (Kwon et al., 2001). The transcription levels of some genes associated with oxidative phosphorylation change upon treatment with Kanamycin B in this study. These observations together raise the possibility that Kanamycin B may regulate the expression with oxidative phosphorylation genes through riboregulatory interactions with the transcripts. Further studies will be required to examine this speculation.

In this study, treatment by Kanamycin B induces transcription of nitrate reductase (*narK*, *narG*, *narI, narH*, and *napA*) and nitrite reductase (*nirB, nirD*, and *nrfA*) genes (Table 1; Supplementary Table S4), which are key genes that are involved in nitrogen metabolism. These reductases participate in the conversion of NO^3−^ to NO^2−^ to NO in cells and are associated with wider essential metabolic processes such as energy production, amino acid metabolism, biofilm formation, antibiotic resistance and bacterial pathogenesis. Nitrogen metabolism is also closely interlinked with biofilm formation that is also linked with the metabolism of glutamic acid, glutamine and arginine. It is widely recognized that NO plays an important role in modulating the architecture of biofilms (Vázquez-Torres and Bäumler, 2016; Rinaldo et al., 2018). Other antibiotics have been reported to be able to induce biofilm formation (Linares et al., 2006). Therefore, it is possible that Kanamycin B may be linked to wider biological functions through its association with nitrogen metabolism.

We have further analyzed the identified DEGs by studying the association and effect of Kanamycin B and the UTR of the DEG gene on reporter gene expression. The result showed that Kanamycin B and other aminoglycosides induced reporter gene expression through the 5′ UTR of the *napF* (involved in nitrogen metabolism) and further direct binding of Kanamycin B to the RNA was detected (Figures 7, 8; Supplementary Figure S1), suggesting that Kanamycin B could directly bind to the 5′ UTR of the *napF* gene to effect expression of the *napF* gene in *E. coli*. Although how the binding of Kanamycin B to the 5′ UTR of the *napF* gene regulate gene expression remains unclear, the result of this finding revealed that Kanamycin B can bind to the UTR RNA of a gene and effect the expression of its downstream gene. In this case, Kanamycin B may effect cellular nitrogen metabolism through binding to the 5′ UTR of the *napF* gene and regulate expression of the gene. Taken together, this study has collectively revealed new insights into the non-antibiotic function of Kanamycin B and its wider cellular effect on *E. coli*.

## Data availability statement

The datasets presented in this study can be found in online repositories. The names of the repository/repositories and accession number(s) can be found in the article/Supplementary material.

## Author contributions

AM and DC wrote the main manuscript text. YC and XZ prepared Figures 1–8; Table 1; Supplementary Tables 1–6; Supplementary Figures S1–S3. All authors contributed to the article and approved the submitted version.

## Funding

This work was supported by National Natural Science Foundation grants 32271345 and 32250710140 to AM.

## Conflict of interest

The authors declare that the research was conducted in the absence of any commercial or financial relationships that could be construed as a potential conflict of interest.

## Publisher’s note

All claims expressed in this article are solely those of the authors and do not necessarily represent those of their affiliated organizations, or those of the publisher, the editors and the reviewers. Any product that may be evaluated in this article, or claim that may be made by its manufacturer, is not guaranteed or endorsed by the publisher.

## Abbreviations

PCA: principal component analysis
DEGs: differentially expressed genes
KEGG: Kyoto Encyclopedia of Genes and Genomes
GO: Gene ontology
STEM: Short Time-series Expression Miner
RT-qPCR: real-time quantitative PCR
*E. coli*: *Escherichia coli*
MST: MicroScale thermophoresis
MIC: minimal inhibitory concentration
