# Supplementary figures and images for: Transcriptome profiling in response to Kanamycin B reveals its wider non-antibiotic cellular function in Escherichia coli
Source: Front Microbiol. 2022 Nov 29;13:937827. doi: 10.3389/fmicb.2022.937827 (PMC9746237; doi:10.3389/fmicb.2022.937827)

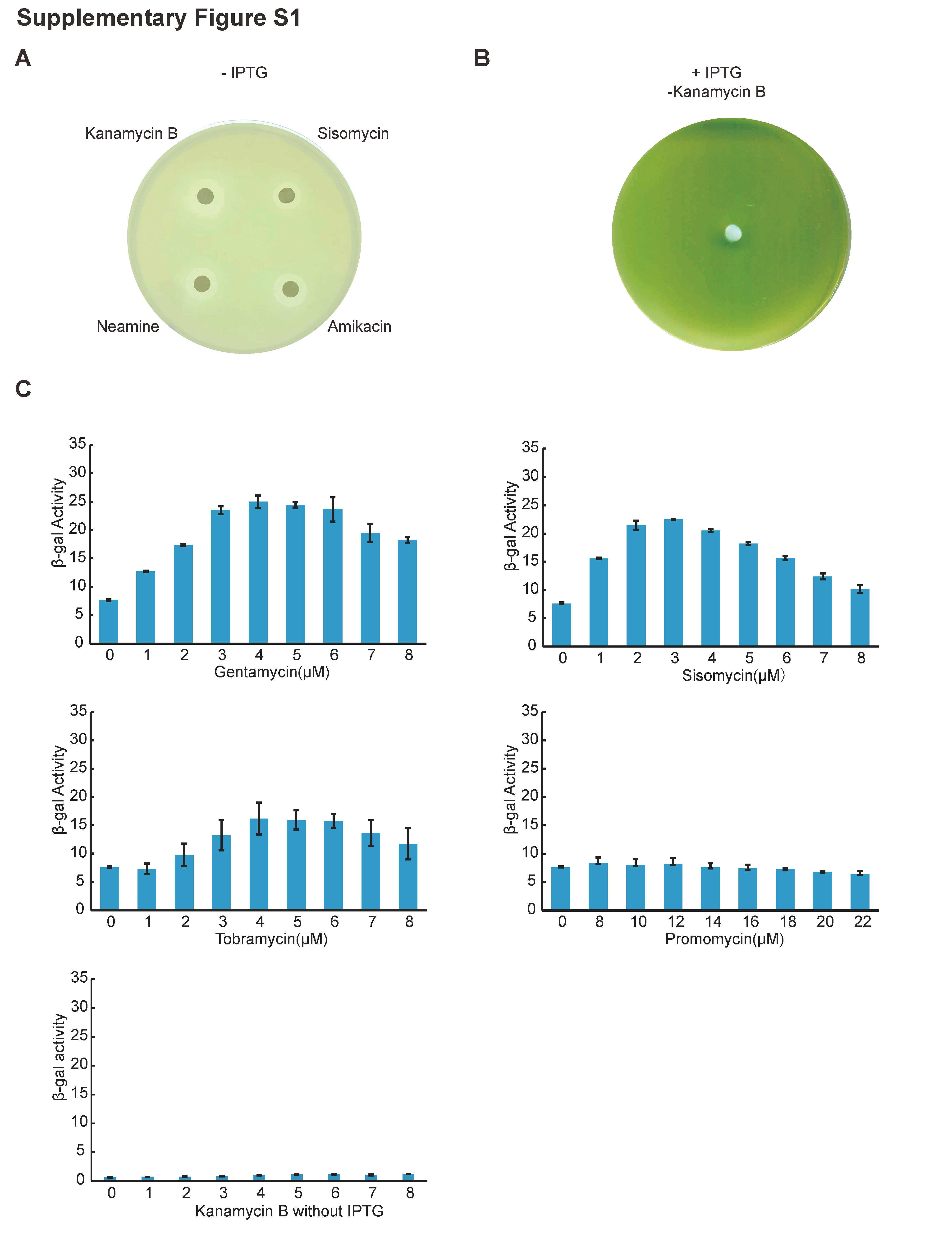

Supplement: Supplementary file 1 [file Image_1.TIF]

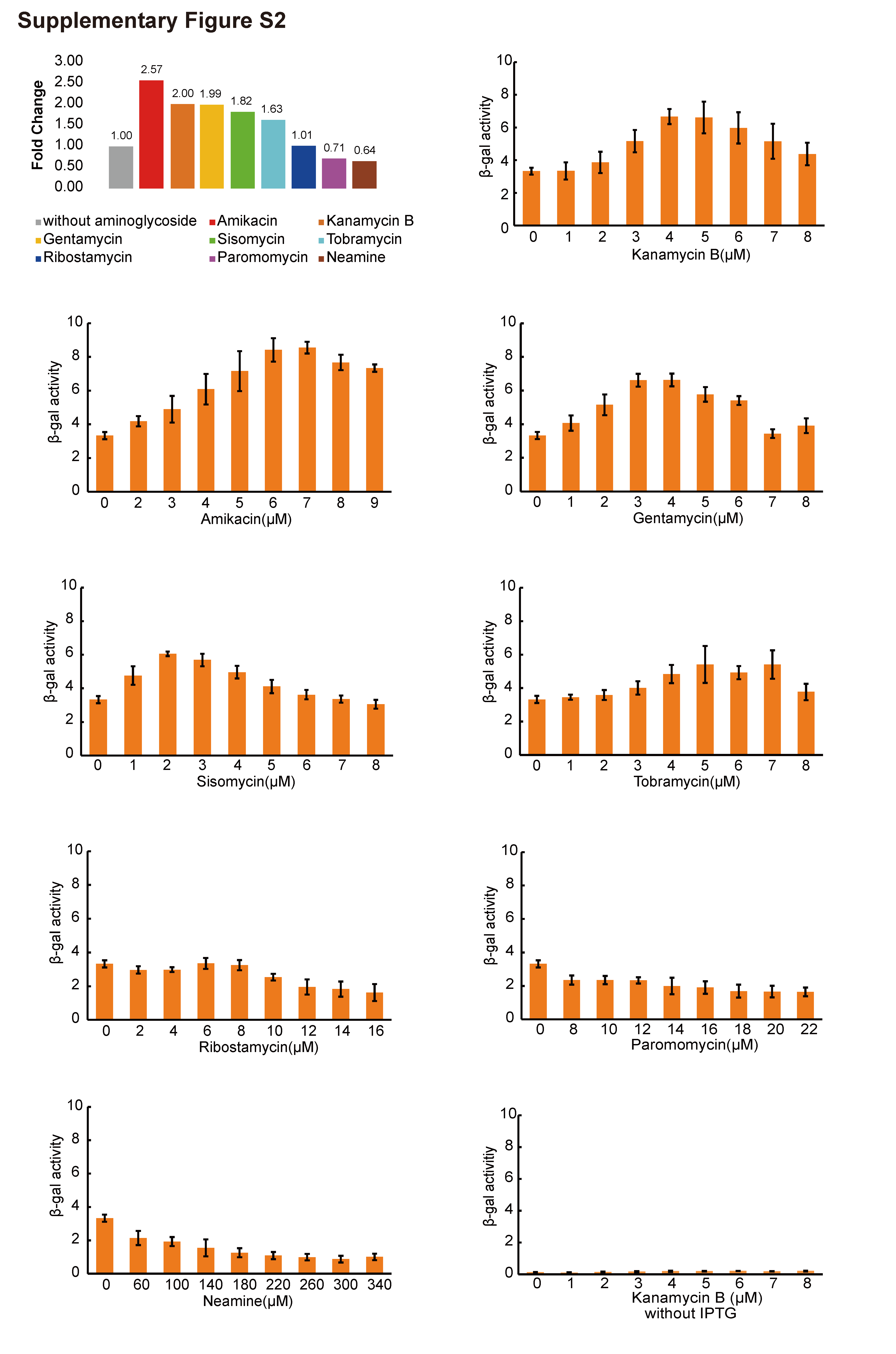

Supplement: Supplementary file 2 [file Image_2.TIF]

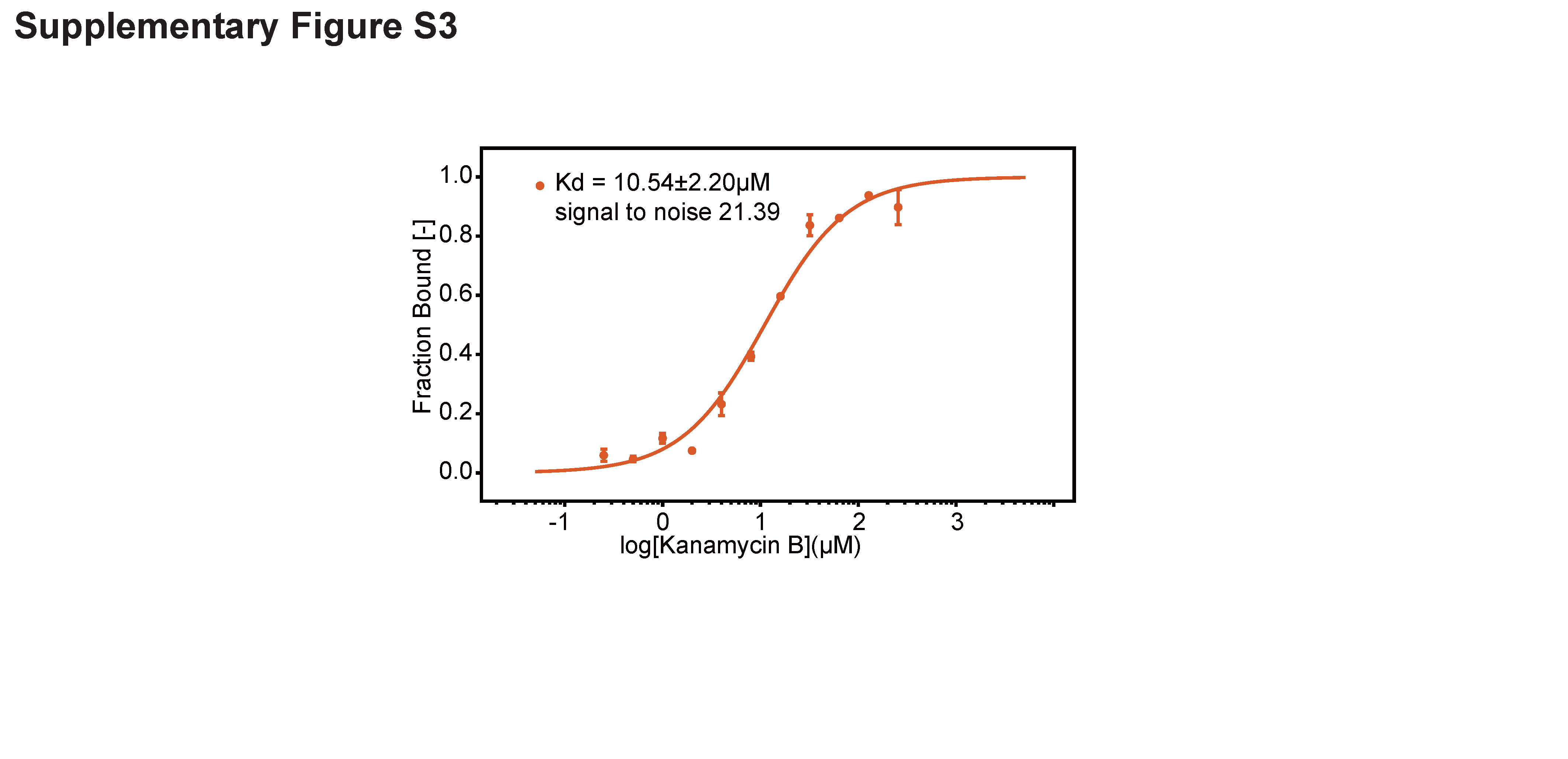

Supplement: Supplementary file 3 [file Image_3.TIF]
