# Supplementary material for: Transcriptome profiling in response to Kanamycin B reveals its wider non-antibiotic cellular function in Escherichia coli
Source: Front Microbiol. 2022 Nov 29;13:937827. doi: 10.3389/fmicb.2022.937827 (PMC9746237; doi:10.3389/fmicb.2022.937827)
Supplement: Supplementary file 4 [file Table_1.DOC]

**Table 1. Functional classification of DEGs (1μM/0μM)**

| **Gene Symbol** | **Fold change** | **Regulation** | **Location** | **Gene annotation** |
| --- | --- | --- | --- | --- |
| **Microbial metabolism in diverse environments** | | | | |
| *nirD** | 219.45 | up | cytosol | nitrite reductase subunit NirD |
| *nirB* | 5.38 | up | cytosol | nitrite reductase catalytic subunit NirB |
| *narI* | 11.30 | up | inner membrane | nitrate reductase A subunit γ |
| *narG* | 9.58 | up | inner membrane | nitrate reductase A subunit α |
| *narH* | 7.48 | up | inner membrane | nitrate reductase A subunit β |
| *ydhX* | 3.61 | up | periplasmic space | putative 4Fe-4S ferredoxin-like protein YdhX |
| *glcD* | 3.09 | up | cytosol | glycolate dehydrogenase, putative FAD-linked subunit |
| *glpE* | 2.75 | up | cytosol | thiosulfate sulfurtransferase GlpE |
| *yqeF* | 2.39 | up | cytosol | putative acyltransferase |
| *lysC* | 2.14 | up | cytosol | aspartate kinase III |
| *frdD* | -9.37 | down | inner membrane | fumarate reductase membrane protein FrdD |
| *frdB* | -9.33 | down | inner membrane, cytosol | fumarate reductase iron-sulfur protein |
| *frdA* | -6.35 | down | inner membrane, cytosol | fumarate reductase flavoprotein subunit |
| *frdC* | -3.00 | down | inner membrane | fumarate reductase membrane protein FrdC |
| *hyaB* | -4.88 | down | periplasmic space, inner membrane | hydrogenase 1 large subunit |
| *hyaA* | -3.41 | down | inner membrane | hydrogenase 1 small subunit |
| *adhE* | -2.86 | down | cytosol | alcohol dehydrogenase/aldehyde-dehydrogenase |
| *adhP* | -2.67 | down | cytosol | ethanol dehydrogenase / alcohol dehydrogenase |
| *gabT* | -3.19 | down | cytosol | 4-aminobutyrate aminotransferase GabT |
| *gabD* | -2.68 | down | cytosol | succinate-semialdehyde dehydrogenase (NADP+) GabD |
| *gadA* | -2.43 | down | cytosol | glutamate decarboxylase A |
| *gadB* | -2.10 | down | cytosol, membrane | glutamate decarboxylase B |
| *hcaE* | -2.86 | down | no annotation | putative 3-phenylpropionate/cinnamate dioxygenase subunit α |
| *paaK* | -2.73 | down | cytosol | phenylacetate-CoA ligase |
| *yghX* | -2.57 | down | no annotation | putative hydrolase fragment |
| *aldB* | -2.43 | down | cytosol | aldehyde dehydrogenase B |
| *fbaB* | -2.19 | down | cytosol | fructose-bisphosphate aldolase class I |
| *tktB* | -2.19 | down | cytosol | transketolase 2 |
| *allB* | -2.16 | down | cytosol | allantoinase |
| **Two-component system** | | | | |
| *fdnG* | 19.98 | up | periplasmic space | formate dehydrogenase N subunit α |
| *fdnH* | 11.67 | up | inner membrane | formate dehydrogenase N subunit β |
| *fdnI* | 5.38 | up | inner membrane | formate dehydrogenase N subunit γ |
| *narI* | 11.30 | up | inner membrane | nitrate reductase A subunit γ |
| *narG* | 9.58 | up | inner membrane | nitrate reductase A subunit α |
| *narH* | 7.48 | up | inner membrane | nitrate reductase A subunit β |
| *narJ* | 6.84 | up | cytosol | nitrate reductase 1 molybdenum cofactor assembly chaperone |
| *narX* | 2.24 | up | periplasmic space, inner membrane | sensory histidine kinase NarX |
| *rstB* | 2.50 | up | inner membrane | sensory histidine kinase RstB |
| *uhpB* | 2.16 | up | inner membrane | sensory histidine kinase UhpB |
| *baeS* | 2.20 | up | inner membrane | sensor histidine kinase BaeS |
| *rcsA* | 3.35 | up | cytosol | DNA-binding transcriptional activator RcsA |
| *ompF* | 2.98 | up | outer membrane | outer membrane porin F |
| *yqeF* | 2.39 | up | cytosol | putative acyltransferase |
| *uhpT* | 2.19 | up | inner membrane | hexose-6-phosphate:phosphate antiporter |
| *frdD* | -9.37 | down | inner membrane | fumarate reductase membrane protein FrdD |
| *frdB* | -9.33 | down | inner membrane, cytosol | fumarate reductase iron-sulfur protein |
| *frdA* | -6.35 | down | inner membrane, cytosol | fumarate reductase flavoprotein subunit |
| *frdC* | -3.00 | down | inner membrane | fumarate reductase membrane protein FrdC |
| *appB* | -6.19 | down | inner membrane | cytochromebd-II ubiquinol oxidase subunit II |
| *appC* | -4.33 | down | inner membrane | cytochromebd-II ubiquinol oxidase subunit I |
| *hyaC* | -7.15 | down | inner membrane | hydrogenase 1 cytochromebsubunit |
| *cusA* | -2.99 | down | inner membrane | copper/silver export system RND permease |
| *mdtC* | -2.58 | down | inner membrane | multidrug efflux pump RND permease subunit MdtC |
| **Butanoate metabolism** | | | |  |
| *yqeF* | 2.39 | up | cytosol | putative acyltransferase |
| *frdD* | -9.37 | down | inner membrane | fumarate reductase membrane protein FrdD |
| *frdB* | -9.33 | down | inner membrane, cytosol | fumarate reductase iron-sulfur protein |
| *frdA* | -6.35 | down | inner membrane, cytosol | fumarate reductase flavoprotein subunit |
| *frdC* | -3.00 | down | inner membrane | fumarate reductase membrane protein FrdC |
| *gabT* | -3.19 | down | cytosol | 4-aminobutyrate aminotransferase GabT |
| *gabD* | -2.68 | down | cytosol | succinate-semialdehyde dehydrogenase (NADP+) GabD |
| *gadA* | -2.43 | down | cytosol | glutamate decarboxylase A |
| *gadB* | -2.10 | down | cytosol, membrane | glutamate decarboxylase B |
| *adhE* | -2.86 | down | cytosol | alcohol dehydrogenase/aldehyde-dehydrogenase |
| *dmlA* | -2.01 | down | cytosol | D-malate/3-isopropylmalate dehydrogenase (decarboxylating) |
| **Nitrogen metabolism** | | | | |
| *nirD** | 219.45 | up | cytosol | nitrite reductase subunit NirD |
| *nirB* | 5.38 | up | cytosol | nitrite reductase catalytic subunit NirB |
| *narK* | 16.98 | up | inner membrane | nitrate:nitrite antiporter NarK |
| *narI* | 11.30 | up | inner membrane | nitrate reductase A subunit γ |
| *narG* | 9.58 | up | inner membrane | nitrate reductase A subunit α |
| *narH* | 7.48 | up | inner membrane | nitrate reductase A subunit β |
| *hcp* | 4.03 | up | cytosol | proteinS-nitrosylase |
| *can* | 2.41 | up | cytosol | carbonic anhydrase 2 |
| **Oxidative phosphorylation** | | | | |
| *cyoA* | 2.48 | up | inner membrane | cytochromebo3ubiquinol oxidase subunit 2 |
| *ppa* | 2.11 | up | cytosol | inorganic pyrophosphatase |
| *frdD* | -9.37 | down | inner membrane | fumarate reductase membrane protein FrdD |
| *frdB* | -9.33 | down | inner membrane, cytosol | fumarate reductase iron-sulfur protein |
| *frdA* | -6.35 | down | inner membrane, cytosol | fumarate reductase flavoprotein subunit |
| *frdC* | -3.00 | down | inner membrane | fumarate reductase membrane protein FrdC |
| *appB* | -6.19 | down | inner membrane | cytochromebd-II ubiquinol oxidase subunit II |
| *appC* | -4.33 | down | inner membrane | cytochromebd-II ubiquinol oxidase subunit I |
| **Alanine, aspartate and glutamate metabolism** | | | | |
| *gabT* | -3.19 | down | cytosol | 4-aminobutyrate aminotransferase GabT |
| *gabD* | -2.68 | down | cytosol | succinate-semialdehyde dehydrogenase (NADP+) GabD |
| *gadA* | -2.43 | down | cytosol | glutamate decarboxylase A |
| *gadB* | -2.10 | down | cytosol, membrane | glutamate decarboxylase B |
| *aspA* | -2.49 | down | cytosol | aspartate ammonia-lyase |
| *glsA* | -2.40 | down | no annotation | glutaminase 1 |
| **Sulfur metabolism** | | | | |
| *ydhX* | 3.61 | up | periplasmic space | putative 4Fe-4S ferredoxin-like protein YdhX |
| *glpE* | 2.75 | up | cytosol | thiosulfate sulfurtransferase GlpE |
| *sbp* | 2.05 | up | periplasmic space | sulfate/thiosulfate ABC transporter periplasmic binding protein Sbp |
| *dmsB* | -4.23 | down | inner membrane | dimethyl sulfoxide reductase subunit B |
| *dmsC* | -3.93 | down | inner membrane | dimethyl sulfoxide reductase subunit C |
| *dmsA* | -2.49 | down | inner membrane | dimethyl sulfoxide reductase subunit A |
| **Arginine and proline metabolism** | | | | |
| *puuD* | 3.81 | up | cytosol | γ-glutamyl-γ-aminobutyrate hydrolase |
| *puuC* | 3.22 | up | cytosol | γ-glutamyl-γ-aminobutyraldehyde dehydrogenase |
| *puuA* | 2.96 | up | cytosol | glutamate-putrescine ligase |
| *speG* | 2.31 | up | cytosol | spermidineN-acetyltransferase |
| *patA* | -2.16 | down | cytosol | putrescine aminotransferase |
| **beta-Alanine metabolism** | | | | |
| *gabT* | -3.19 | down | cytosol | 4-aminobutyrate aminotransferase GabT |
| *gabD* | -2.68 | down | cytosol | succinate-semialdehyde dehydrogenase (NADP+) GabD |
| *gadA* | -2.43 | down | cytosol | glutamate decarboxylase A |
| *gadB* | -2.10 | down | cytosol, membrane | glutamate decarboxylase B |
| **Selenocompound metabolism** | | | | |
| *ynfE* | -9.15 | down | periplasmic space, inner membrane | putative selenate reductase YnfE |
| *ynfF* | -5.26 | down | periplasmic space, inner membrane | putative selenate reductase YnfF |
| *sufS* | -2.31 | down | cytosol | L-cysteine desulfurase |
| metE | -2.15 | down | cytosol | cobalamin-independent homocysteine transmethylase |
| **Tyrosine metabolism** | | | | |
| *adhE* | -2.86 | down | cytosol | alcohol dehydrogenase/aldehyde-dehydrogenase |
| *adhP* | -2.67 | down | cytosol | ethanol dehydrogenase / alcohol dehydrogenase |
| gabD | -2.68 | down | cytosol | succinate-semialdehyde dehydrogenase (NADP+) GabD |
| **Chloroalkane and chloroalkene degradation** | | | | |
| *adhE* | -2.86 | down | cytosol | alcohol dehydrogenase/aldehyde-dehydrogenase |
| *adhP* | -2.67 | down | cytosol | ethanol dehydrogenase / alcohol dehydrogenase |
| **Naphthalene degradation** | | | | |
| *adhE* | -2.86 | down | cytosol | alcohol dehydrogenase/aldehyde-dehydrogenase |
| *adhP* | -2.67 | down | cytosol | ethanol dehydrogenase / alcohol dehydrogenase |
| **Nitrotoluene degradation** | | | | |
| *hyaB* | -4.88 | down | periplasmic space, inner membrane | hydrogenase 1 large subunit |
| *hyaA* | -3.41 | down | inner membrane | hydrogenase 1 small subunit |
| **Polyketide sugar unit biosynthesis** | | | | |
| *rfbD* | 2.97 | up | cytosol | dTDP-4-dehydrorhamnose reductase |
| *rfbA* | 2.48 | up | cytosol | dTDP-glucose pyrophosphorylase |
| **Taurine and hypotaurine metabolism** | | | | |
| *gadA* | -2.43 | down | cytosol | glutamate decarboxylase A |
| **Chlorocyclohexane and chlorobenzene degradation** | | | |  |
| *yghX* | -2.57 | down | no annotation | putative hydrolase fragment |
| *gadB* | -2.10 | down | cytosol, membrane | glutamate decarboxylase B |
| **Fluorobenzoate degradation** | | | | |
| *yghX* | -2.57 | down | no annotation | putative hydrolase fragment |
| **Inositol phosphate metabolism** | | | | |
| *appA* | -3.96 | down | periplasmic space | periplasmic phosphoanhydride phosphatase/multiple inositol-polyphosphate phosphatase |
| **Toluene degradation** | | | | |
| *yghX* | -2.57 | down | no annotation | putative hydrolase fragment |
| **Biosynthesis of ansamycins** | | | | |
| *tktB* | -2.19 | down | cytosol | transketolase 2 |
| **Synthesis and degradation of ketone bodies** | | | | |
| *yqeF* | 2.39 | up | cytosol | putative acyltransferase |

* The counts of the nirD gene is 0.
