# Supplementary material for: Transcriptome profiling in response to Kanamycin B reveals its wider non-antibiotic cellular function in Escherichia coli
Source: Front Microbiol. 2022 Nov 29;13:937827. doi: 10.3389/fmicb.2022.937827 (PMC9746237; doi:10.3389/fmicb.2022.937827)
Supplement: Supplementary file 5 [file Table_3.DOC]

**Supplementary Table S1.** List of randomly selected DEGs for RT-qPCR.

| Gene Name | Primer sequenses |
| --- | --- |
| narK F | GCGTGGTTTGGCATGAACG |
| narK R | AAGCCGATGAAGGAGCCGAA |
| yfcV F | ATGTTAGCCTGGCGATCGG |
| yfcV R | GAGTCTGCTTCGCTTTGCC |
| narH F | ATGTATTTGCCGCGCCTGTG |
| narH R | CGCTTGTAAATCGCACCGC |
| napF F | TCGCTATTTTCTCCGCGCC |
| napF R | GGCGGCATTCAACTGACTG |
| grcA F | TGCGTCGCGAAACTCTGGAA |
| grcA R | ATAACGTCGCGCTGCTGTTC |
| gadC F | AGCGCTGAATGAAGACCC |
| gadC R | TTCGCGCCGTGTATTTCG |
| frdA F | AGAGCTGCAGGAACGCTTC |
| frdA R | AGACCGTGGCCCAGTTCAA |
| ompW F | AGAAGGTGCTGGTGGTACG |
| ompW R | GCGTCGCTGCCAGTAATTCC |
| absolute qPCR amp F | AGTATTCAACATTTCCGTGTCGCCC |
| absolute qPCR amp R | GCTAGAGTAAGTAGTTCGCC |
