# Supplementary material for: Transcriptome profiling in response to Kanamycin B reveals its wider non-antibiotic cellular function in Escherichia coli
Source: Front Microbiol. 2022 Nov 29;13:937827. doi: 10.3389/fmicb.2022.937827 (PMC9746237; doi:10.3389/fmicb.2022.937827)
Supplement: Supplementary file 8 [file Table_6.DOCX]

**Supplementary Table S4. Functional classification of DEGs (0.5μM-0μM)**

| **Gene Symbol** | **Fold change** | **Regulation** | **location** | **Gene annotation** |
| --- | --- | --- | --- | --- |
| **Microbial metabolism in diverse environments** | | | |  |
| *nirD** | 262.33 | up | cytosol | nitrite reductase subunit NirD |
| *nirB* | 5.96 | up | cytosol | nitrite reductase catalytic subunit NirB |
| *narG* | 10.54 | up | inner membrane | nitrate reductase A subunit α |
| *narH* | 7.15 | up | inner membrane | nitrate reductase A subunit β |
| *narI* | 12.58 | up | inner membrane | nitrate reductase A subunit γ |
| *glcE* | 3.34 | up | no annotation | glycolate dehydrogenase, putative FAD-binding subunit |
| *glcD* | 2.54 | up | cytosol | glycolate dehydrogenase, putative FAD-linked subunit |
| *napA* | 2.15 | up | periplasmic space | periplasmic nitrate reductase subunit NapA |
| *nrfA* | 2.08 | up | periplasmic space | cytochromec552nitrite reductase |
| *xdhA* | 2.22 | up | no annotation | putative xanthine dehydrogenase molybdenum-binding subunit XdhA |
| *hcaB** | -25.00 | down | cytosol | 2,3-dihydroxy-2,3-dihydrophenylpropionate dehydrogenase |
| *hcaE* | -2.00 | down | no annotation | putative 3-phenylpropionate/cinnamate dioxygenase subunit α |
| *frdC* | -7.69 | down | inner membrane | fumarate reductase membrane protein FrdC |
| *frdB* | -5.56 | down | inner membrane, cytosol | fumarate reductase iron-sulfur protein |
| *frdD* | -3.13 | down | inner membrane | fumarate reductase membrane protein FrdD |
| *frdA* | -2.94 | down | inner membrane, cytosol | fumarate reductase flavoprotein subunit |
| *adhP* | -2.63 | down | cytosol | ethanol dehydrogenase / alcohol dehydrogenase |
| *adhE* | -2.17 | down | cytosol | alcohol dehydrogenase/aldehyde-dehydrogenase |
| *allC* | -2.63 | down | cytosol | allantoate amidohydrolase |
| *pdeH* | -2.44 | down | cytosol | c-di-GMP phosphodiesterase PdeH |
| *yghX* | -2.08 | down | no annotation | putative hydrolase fragment |
| **Two-component system** | |  |  |  |
| *fdnG* | 19.77 | up | periplasmic space | formate dehydrogenase N subunit α |
| *fdnH* | 11.28 | up | inner membrane | formate dehydrogenase N subunit β |
| *fdnI* | 5.83 | up | inner membrane | formate dehydrogenase N subunit γ |
| *narI* | 12.58 | up | inner membrane | nitrate reductase A subunit γ |
| *narG* | 10.54 | up | inner membrane | nitrate reductase A subunit α |
| *narH* | 7.15 | up | inner membrane | nitrate reductase A subunit β |
| *narJ* | 7.46 | up | cytosol | nitrate reductase 1 molybdenum cofactor assembly chaperone |
| *narL* | 3.02 | up | cytosol | DNA-binding transcriptional dual regulator NarL |
| *rcsA* | 2.69 | up | cytosol | DNA-binding transcriptional activator RcsA |
| *zraS* | 2.42 | up | inner membrane | sensor histidine kinase ZraS |
| *frdC* | -7.69 | down | inner membrane | fumarate reductase membrane protein FrdC |
| *frdB* | -5.56 | down | inner membrane, cytosol | fumarate reductase iron-sulfur protein |
| *frdD* | -3.13 | down | inner membrane | fumarate reductase membrane protein FrdD |
| *frdA* | -2.94 | down | inner membrane, cytosol | fumarate reductase flavoprotein subunit |
| *appC* | -3.03 | down | inner membrane | cytochromebd-II ubiquinol oxidase subunit I |
| *appB* | -2.94 | down | inner membrane | cytochromebd-II ubiquinol oxidase subunit II |
| *cheW* | -4.76 | down | inner membrane, cytosol | chemotaxis protein CheW |
| *cusS* | -2.50 | down | inner membrane, cytosol, periplasmic space | sensor histidine kinase CusS |
| *dpiB* | -2.17 | down | periplasmic space, cytosol, inner membrane | sensor histidine kinase DpiB |
| **Butanoate metabolism** | |  |  |  |
| *frdC* | -7.69 | down | inner membrane | fumarate reductase membrane protein FrdC |
| *frdB* | -5.56 | down | inner membrane, cytosol | fumarate reductase iron-sulfur protein |
| *frdD* | -3.13 | down | inner membrane | fumarate reductase membrane protein FrdD |
| *frdA* | -2.94 | down | inner membrane, cytosol | fumarate reductase flavoprotein subunit |
| *adhE* | -2.17 | down | cytosol | alcohol dehydrogenase/aldehyde-dehydrogenase |
| **Nitrogen metabolism** | |  |  |  |
| *nirD** | 262.33 | up | cytosol | nitrite reductase subunit NirD |
| *nirB* | 5.96 | up | cytosol | nitrite reductase catalytic subunit NirB |
| *narK* | 16.2 | up | inner membrane | nitrate:nitrite antiporter NarK |
| *narI* | 12.58 | up | inner membrane | nitrate reductase A subunit γ |
| *narG* | 10.54 | up | inner membrane | nitrate reductase A subunit α |
| *narH* | 7.15 | up | inner membrane | nitrate reductase A subunit β |
| *hcp* | 4.35 | up | cytosol | proteinS-nitrosylase |
| *napA* | 2.15 | up | periplasmic space | periplasmic nitrate reductase subunit NapA |
| *nrfA* | 2.08 | up | periplasmic space | cytochromec552nitrite reductase |
| **Oxidative phosphorylation** | |  |  |  |
| *frdC* | -7.69 | down | inner membrane | fumarate reductase membrane protein FrdC |
| *frdB* | -5.56 | down | inner membrane, cytosol | fumarate reductase iron-sulfur protein |
| *frdD* | -3.13 | down | inner membrane | fumarate reductase membrane protein FrdD |
| *frdA* | -2.94 | down | inner membrane, cytosol | fumarate reductase flavoprotein subunit |
| *appC* | -3.03 | down | inner membrane | cytochromebd-II ubiquinol oxidase subunit I |
| *appB* | -2.94 | down | inner membrane | cytochromebd-II ubiquinol oxidase subunit II |
| **Arginine and proline metabolism** | | |  |  |
| *puuB* | 10.09 | up | cytosol | γ-glutamylputrescine oxidase |
| *puuC* | 4.79 | up | cytosol | γ-glutamyl-γ-aminobutyraldehyde dehydrogenase |
| *puuD* | 3.08 | up | cytosol | γ-glutamyl-γ-aminobutyrate hydrolase |
| *puuA* | 2.71 | up | cytosol | glutamate-putrescine ligase |
| *patD* | -2.27 | down | cytosol | γ-aminobutyraldehyde dehydrogenase |
| **Tyrosine metabolism** | |  |  |  |
| *adhP* | -2.63 | down | cytosol | ethanol dehydrogenase / alcohol dehydrogenase |
| *adhE* | -2.17 | down | cytosol | alcohol dehydrogenase/aldehyde-dehydrogenase |
| **Chloroalkane and chloroalkene degradation** | | |  |  |
| *adhP* | -2.63 | down | cytosol | ethanol dehydrogenase / alcohol dehydrogenase |
| *adhE* | -2.17 | down | cytosol | alcohol dehydrogenase/aldehyde-dehydrogenase |
| **Naphthalene degradation** | |  |  |  |
| *adhP* | -2.63 | down | cytosol | ethanol dehydrogenase / alcohol dehydrogenase |
| *adhE* | -2.17 | down | cytosol | alcohol dehydrogenase/aldehyde-dehydrogenase |
| **Chlorocyclohexane and chlorobenzene degradation** | | | | |
| *yghX* | -2.08 | down | no annotation | putative hydrolase fragment |
| **Fluorobenzoate degradation** | |  |  |  |
| *yghX* | -2.08 | down | no annotation | putative hydrolase fragment |
| **Inositol phosphate metabolism** | | | |  |
| *appA* | -2.78 | down | periplasmic space | periplasmic phosphoanhydride phosphatase/multiple inositol-polyphosphate phosphatase |
| **Toluene degradation** | |  |  |  |
| *yghX* | -2.08 | down | no annotation | putative hydrolase fragment |
| **Aminoacyl-tRNA biosynthesis** | | | |  |
| *lysQ* | 7.56 | up | cytosol | tRNA-Lys(UUU) |
| *leuX* | 2.33 | up | cytosol | tRNA-Leu(CAA) |
| *valU* | -4.00 | down | cytosol | tRNA-Val(UAC) |
| *lysT* | -3.85 | down | cytosol | tRNA-Lys(UUU) |
| *valZ* | -3.45 | down | cytosol | tRNA-Val(UAC) |
| *leuQ* | -2.70 | down | cytosol | tRNA-Leu(CAG) |
| *glyW* | -2.63 | down | cytosol | tRNA-Gly(GCC) |
| *metZ* | -2.33 | down | cytosol | tRNA-initiator Met(CAU) |
| *valX* | -2.33 | down | cytosol | tRNA-Val(UAC) |
| *gltT* | -2.08 | down | cytosol | tRNA-Glu(UUC) |
| *serV* | -2.04 | down | cytosol | tRNA-Ser(GCU) |
| **Citrate cycle (TCA cycle)** | |  |  |  |
| *frdC* | -7.69 | down | inner membrane | fumarate reductase membrane protein FrdC |
| *frdB* | -5.56 | down | inner membrane, cytosol | fumarate reductase iron-sulfur protein |
| *frdD* | -3.13 | down | inner membrane | fumarate reductase membrane protein FrdD |
| *frdA* | -2.94 | down | inner membrane, cytosol | fumarate reductase flavoprotein subunit |
| **Degradation of aromatic compounds** | | |  |  |
| *hcaB** | -25.00 | down | cytosol | 2,3-dihydroxy-2,3-dihydrophenylpropionate dehydrogenase |
| *hcaE* | -2.00 | down | no annotation | putative 3-phenylpropionate/cinnamate dioxygenase subunit α |
| *adhP* | -2.63 | down | cytosol | ethanol dehydrogenase / alcohol dehydrogenase |
| *adhE* | -2.17 | down | cytosol | alcohol dehydrogenase/aldehyde-dehydrogenase |
| **Salmonella infection** | |  |  |  |
| *norR* | 2.29 | up | cytosol | DNA-binding transcriptional dual regulator NorR |
| *nrfA* | 2.08 | up | periplasmic space | cytochromec552nitrite reductase |

* The counts of the *nirD* and *hcaB* gene is 0.
